# Risk of acute myocardial infarction during use of individual NSAIDs: A nested case-control study from the SOS project

**DOI:** 10.1371/journal.pone.0204746

**Published:** 2018-11-01

**Authors:** Gwen M. C. Masclee, Huub Straatman, Andrea Arfè, Jordi Castellsague, Edeltraut Garbe, Ron Herings, Bianca Kollhorst, Silvia Lucchi, Susana Perez-Gutthann, Silvana Romio, René Schade, Tania Schink, Martijn J. Schuemie, Lorenza Scotti, Cristina Varas-Lorenzo, Vera E. Valkhoff, Marco Villa, Miriam C. J. M. Sturkenboom

**Affiliations:** 1 Department of Gastroenterology and Hepatology, Deventer Hospital, Deventer, the Netherlands; 2 PHARMO Institute, Utrecht, The Netherlands; 3 Unit of Biostatistics, Epidemiology and Public Health, Department of Statistics and Quantitative Methods, University Milano-Bicocca, Milano, Italy; 4 RTI Health Solutions, Barcelona, Spain; 5 Leibniz Institute of Prevention Research and Epidemiology, Bremen, Germany; 6 Local Health Authority ASL Cremona, Cremona, Italy; 7 Department of Medical Informatics, Erasmus University Medical Center, Rotterdam, The Netherlands; 8 Department of Gastroenterology and Hepatology, Erasmus University Medical Center, Rotterdam, The Netherlands; 9 Julius Global Health, Utrecht, The Netherlands; University of Catanzaro, ITALY

## Abstract

**Background:**

Use of selective COX-2 non-steroidal anti-inflammatory drugs (NSAIDs) (coxibs) has been associated with an increased risk of acute myocardial infarction (AMI). However, the risk of AMI has only been studied for very few NSAIDs that are frequently used.

**Objectives:**

To estimate the risk of AMI for individual NSAIDs.

**Methods:**

A nested case-control study was performed from a cohort of new NSAID users ≥18 years (1999–2011) matching cases to a maximum of 100 controls on database, sex, age, and calendar time. Data were retrieved from six healthcare databases. Adjusted odds ratios (ORs) of current use of individual NSAIDs compared to past use were estimated per database. Pooling was done by two-stage pooling using a random effects model (ORmeta) and by one-stage pooling (ORpool).

**Results:**

Among 8.5 million new NSAID users, 79,553 AMI cases were identified. The risk was elevated for current use of ketorolac (ORmeta 2.06;95%CI 1.83–2.32, ORpool 1.80; 1.49–2.18) followed, in descending order of point estimate, by indometacin, etoricoxib, rofecoxib, diclofenac, fixed combination of diclofenac with misoprostol, piroxicam, ibuprofen, naproxen, celecoxib, meloxicam, nimesulide and ketoprofen (ORmeta 1.12; 1.03–1.22, ORpool 1.00;0.86–1.16). Higher doses showed higher risk estimates than lower doses.

**Conclusions:**

The relative risk estimates of AMI differed slightly between 28 individual NSAIDs. The relative risk was highest for ketorolac and was correlated with COX-2 potency, but not restricted to coxibs.

## Introduction

Non-steroidal anti-inflammatory drugs (NSAIDs) are widely used to reduce inflammation and provide pain relief. They act via reversible, competitive inhibition of cyclo-oxygenase (COX) enzymes. As inhibition of the COX-1 enzyme decreases prostaglandins production, gastrointestinal adverse events including ulcerations and bleeding occur often during NSAID use. This led to development of selective COX-2 inhibitors (coxibs) which after successful market introduction [1,2] were considered with cardiovascular safety resulting in the voluntary withdrawal of rofecoxib in 2004.[3] The underlying mechanism of increased cardiovascular events may be related to a dysbalance in COX-1 and COX-2 inhibition properties favoring thrombosis by vasoconstriction and platelet aggregation.[4,5]

Reviews by the United States Food and Drug Administration and the European Medicines Agency (EMA) concluded that coxibs increase the risk of CV events.[6,7] It was recommended, for instance by the American Heart Association, in 2005 to avoid the use of coxibs in patients with ischemic heart disease, stroke or peripheral arterial disease.[7–10] At that moment little information was available about the CV risk of NSAIDs, but further studies showed signals of increased arterial thrombosis risk for non-selective (ns) NSAIDs, particularly when used in high doses and for long-term.[4,11] Based on the uncertainty, EMA requested a review of the CV safety of nsNSAIDs as well, which resulted in the Non-Steroidal Anti-inflammatory Drugs (SOS) project.[12] Previously published meta-analyses of clinical trials[13] or from observational studies reported on increased risks of composite cardiovascular endpoints for only a few traditional NSAIDs and coxibs.[14] This SOS study aimed to assess and summarize the risk of acute myocardial infarction (AMI) associated with the use of a large variety of individual NSAIDs in Europe.

## Material and methods

### Study design and data sources

A nested case-control study was conducted within a cohort of new NSAID users during the study period.

Data for this study was obtained from six different longitudinal population-based health care databases from four European countries (GePaRD from Germany (GE), OSSIFF and SISR from Italy (IT), IPCI and PHARMO from the Netherlands (NL) and THIN from the United Kingdom (UK)) covering a source population of around 32 million subjects. All databases have been used for pharmacoepidemiological research (S1 Table)[15–18] and are described detailed in previous publications.[19,20]

The German Pharmacoepidemiological Research Database (GePaRD) is a database comprising data from five statutory health insurance companies throughout Germany and currently covers around 14 million persons representing approximately 20% of the population.[17] The Health Improvement Network (THIN) database is a general practice (GP) database in the UK and currently captures medical records of 11.1 million patients.[16,21] The Integrated Primary Care Information (IPCI) database is a GP database from the Netherlands and currently covers over 1.5 million people [18], PHARMO database is a medical record linkage system of 2.2 million community-dwelling inhabitants in the Netherlands.[15] OSSIFF (Osservatorio Interaziendale per la Farmacoepidemiologia e la Farmacoeconomia) is a database capturing national health service data and clinical registries from several local health agencies in Lombardy) for a population of about 2.9 million people. The second Italian database SISR (Sistema Informativo Sanitario Regionale) obtains national health service data from the Lombardy region, with about nine million inhabitants (approximately 16% of the national population). To avoid overlap the OSSIFF population was removed from the SISR database.

All GP and claims databases contain information on demographics of the population, diagnoses (in- and/or outpatient), and drug prescriptions/dispensings. The diagnoses captured by the databases are coded with four disease coding systems including the International Classification of Diseases (ICD) 9^th^ or 10^th^ revision [22], International Classification for Primary Care (ICPC)[23], or READ.[24] Mapping of concepts and codes was performed using the Unified Medical Language System (UMLS), according to a previously described workflow.[25,26] All drugs were mapped to the World Health Organization’s (WHO) Anatomical Therapeutic Chemical classification.[27] A distributed approach was used for collaboration: all database custodians extracted data locally; original data were transformed into a common data model; codes for outcome and covariates were mapped using an extensive harmonization strategy; and a common standardized script (Jerboa, Erasmus University Medical Center)[28] was used to create aggregated tables that were subsequently encrypted and shared on a central data warehouse for further analysis. [19,20,28]

### Study cohort

In each database, we identified a cohort of patients aged≥18 years who received at least one new NSAID prescription (S2 Table) during the database-specific study period within the general study period which started 1 January 1999 and ended 31 December 2011 (S1 Table).

Cohort entry was the date of first NSAID prescription/dispensation during the study period. Patients were excluded if they received any NSAID prescription in the year before to avoid prevalent user bias.[29] Patients needed to have at least one year of continuous database history to allow assessment of confounders and exclusion criteria. All subjects with a cancer diagnosis (except non-melanoma skincancer) during the one year preceding cohort entry were excluded from the cohort. All NSAID cohort members were followed from the date of cohort entry until date of AMI diagnosis, cancer, death, last data supply, transferring out of the database, or end of the study period, whichever was earliest.

### Cases and controls

The outcome was a first hospitalization with a discharge diagnosis code of AMI (GePaRD, PHARMO, OSSIFF, and SISR) or a first diagnosis of AMI (THIN and IPCI) during follow-up (S3 Table for codes). In OSSIFF a sample of hospitalization codes were validated against the hospitalization records (PPV 89%; 95%CI: 77–96). In IPCI all cases were validated. PPVs of codes used to identify AMI in the current study ranged between 95% (ICD-9CM) and 100% (ICD-10) in previous studies.[30,31] The date of recorded diagnosis or admission date of AMI was used as index date. Within each database, up to 100 controls were matched on the index date (‘event’ date) to each case by risk set sampling on age (± 1 year), sex and cohort entry (± 28 days).

### NSAID-exposure

Exposure to individual NSAIDs was obtained from prescriptions or from outpatient drug dispensings claims. Duration was obtained by dividing the total units by the daily number of units prescribed (THIN, IPCI, and PHARMO: prescribed duration), for other databases standard durations were used based on the country specific defined daily dose (DDD) values.[32] Mean duration of NSAID use in the cohort is shown in S8 Table.

Recency of exposure to individual NSAIDs was classified using the interval between index date and the end of the most recent NSAID use before the index date: 1) ≤14 days before index date classified as ‘current’ use; 2) between 15 and 183 days before index date as ‘recent’ and; 3) ≥184 days before index date as ‘past’. Exposure periods were considered mutually exclusive. Duration of current use was then classified into very short (1–6 days), short (7–29), medium (30–89) and long (≥ 90). If multiple NSAIDs were used in the current period, NSAID use was assigned to current use of all NSAIDs. We assigned current exposure to the most recent NSAID used prior to index date if patients switched between NSAIDs. Past use of any NSAID was considered as common reference group in order to compare across NSAIDs.

In IPCI, THIN and PHARMO daily dose of NSAID was estimated from the prescribing regimen and strength. Dose of current exposure to each individual NSAID was classified using the ratio of prescribed daily dose (PDD) compared to DDD in order to allow for comparison across NSAIDs, daily dose was categorized as low dose (<0.8 PDD/DDD), normal dose (0.8–1.2 PDD/DDD) and high dose (≥1.3 PDD/DDD) (S4 Table).

### Covariates

Covariates were classified into a-priori risk factors (history of ischemic heart disease (excluding AMI); history of stroke; heart failure; diabetes mellitus type 2; hyperlipidemia; smoking; use of ACE inhibitors, antithrombotic agents, low-dose aspirin, beta blockers, calcium channel blockers, diuretics, glucocorticoids, nitrates, oral contraceptives, platelet aggregation inhibitors, lipid lowering drugs and postmenopausal hormone therapy) or potential confounders (obesity, osteoarthritis and use of anticoagulants). They were measured during the 12 months prior to cohort entry or in 30 or 90 days before index date.

### Statistical analyses

To estimate the risk for AMI among current use of an individual NSAID in comparison to past use of any NSAID, odds ratios were calculated using conditional logistic regression analyses for each database separately if five or more exposed cases per database were available. Combining data from several sources is possible in mainly two ways: 1) one-stage analysis; which consists of performing the analysis on one large database where individual patient-level data from different databases is pooled; or 2) two stage meta-analysis; in which the analyses are performed on each single database and the summary statistics are combined using standard meta-analysis techniques. Two stage pooled NSAID-specific ORs (ORmeta) were calculated both using fixed and random effects meta-analysis.[33,34] The degree of statistical heterogeneity was measured by I^2^.[35] One stage pooling of data across databases was performed by combining the matched case control sets and analysis with conditional logistic regression adjusted for covariates. This approach has most power and provides one overall risk measure (ORpooled) for all NSAIDs with at least five exposed cases.

A stepwise approach was used for confounder selection in both approaches: 1) a-priori selected confounders were always included; 2) univariate analyses for each potential confounder with a prevalence of 5% in controls, which were added to the model if Wald *p*-value was <0.05; 3) backward selection of potential confounders (*p*-value>0.05).

Categorical duration analyses were performed within current users of each individual NSAID, using short duration (7–29 days) as reference group. Dose analyses were done by categories comparing dose levels to past use of any NSAID and by continuous analyses through restricted cubic splines (3 knots) and through fractional polynomial regression (maximum of 2 terms).[36] Since potency of COX-inhibition of NSAIDs is based on normal therapeutic doses, the relationship between COX-2 potency and the relative risk of AMI was visualized by plotting the relative risk results by the COX-2 inhibition using normal daily doses (PDD/DDD between 0.8 and 1.2). [4]

Effect modification was investigated by stratification for sex, age (≤60 or >60 years), prior ischemic heart disease, use of aspirin or lipid lowering drugs and calendar year (≤2004 or >2004), we tested for multiplicative interaction. All analyses were performed using SAS (Cary, NC version 9.2).

## Results

The study cohort comprised 8,535,952 new NSAID users (S1 Fig), of whom 101,227 patients developed an AMI after cohort entry. Of these, 79,553 (78.6%) cases could be matched to at least one control. Baseline characteristics of cases and matched controls are shown in Table 1 and exposure in Table 2. If persons used multiple NSAIDs each individual NSAID was considered; 93–97% of current users used only one NSAID. Cases had more often risk factors for AMI such as a prior history of ischemic heart disease, other cardiovascular diseases or use of cardiovascular drugs, than controls but the prevalence of these risk factors did not differ between different NSAID users (S6 Table).

**Table 1:** Characteristics of AMI cases and matched controls by database.

	Germany		The Netherlands				Italy				United Kingdom	
	**GePaRD**		**IPCI**		**PHARMO**		**SISR**		**OSSIFF**		**THIN**	
	**Cases**	**Controls**	**Cases**	**Controls**	**Cases**	**Controls**	**Cases**	**Controls**	**Cases**	**Controls**	**Cases**	**Controls**
	**N = 9,930**	**N = 957,016**	**N = 1,070**	**N = 38,688**	**N = 9,974**	**N = 896,907**	**N = 25,719**	**N = 2,523,118**	**N = 19,349**	**N = 1,840,368**	**N = 13,511**	**N = 1,232,506**
	**N (%)**	**N (%)**	**N (%)**	**N (%)**	**N (%)**	**N (%)**	**N (%)**	**N (%)**	**N (%)**	**N (%)**	**N (%)**	**N (%)**
Age in years (mean±sd)*	62.4 (12.4)	62.0 (11.8)	63.3 (13.6)	58.7 (11.3)	61.0 (13.7)	59.3 (12.9)	69.8 (12.2)	69.5 (12.1)	67.7 (12.2)	67.0 (11.8)	64.3 (13.3)	63.1 (12.5)
Sex*												
Male	7,694 (77.5)	747,457 (78.1)	678 (63.4)	24,661 (63.7)	6,526 (65.4)	586,796 (65.4)	14,069 (54.7)	1,377,184 (54.6)	10,939 (56.5)	1,036,481 (56.3)	8,473 (62.7)	774,360 (62.8)
Female	2,236 (22.5)	209,559 (21.9)	392 (36.6)	14,027 (36.3)	3,448 (34.6)	310,111 (34.6)	11,650 (45.3)	1,145,934 (45.4)	8,410 (43.5)	803,887 (43.7)	5,038 (37.3)	458,146 (37.2)
A-priori confounders**												
Diabetes mellitus type 2	1612 (16.23)	81139 (8.48)	82 (7.66)	1453 (3.76)	1041 (10.44)	52421 (5.84)	5485 (21.33)	261807 (10.38)	2912 (15.05)	124145 (6.75)	1231 (9.11)	60056 (4.87)
Heart Failure	1244 (12.53)	68847 (7.19)	36 (3.36)	581 (1.50)	70 (0.70)	3264 (0.36)	1520 (5.91)	78461 (3.11)	831 (4.29)	37932 (2.06)	197 (1.46)	6440 (0.52)
Hyperlipidemia	2593 (26.11)	69643 (17.73)	135 (12.62)	3438 (8.89)	1695 (16.99)	104860 (11.69)	5834 (22.68)	404273 (16.02)	2819 (14.57)	172089 (9.35)	2495 (18.47)	151437 (12.29)
Ischemic Heart Disease	2877 (28.97)	152110 (15.89)	49 (4.58)	856 (2.21)	176 (1.76)	8068 (0.90)	1417 (5.51)	48222 (1.91)	895 (4.63)	29902 (1.62)	695 (5.14)	28136 (2.28)
Current Smoking	NA	NA	187 (17.48)	7179 (18.56)	NA	NA	NA	NA	NA	NA	1518 (11.24)	88754 (7.20)
Stroke	683 (6.88)	38068 (3.98)	26 (2.43)	678 (1.75)	14 (0.14)	602 (0.07)	252 (0.98)	12994 (0.51)	218 (1.13)	10241 (0.56)	70 (0.52)	4181 (0.34)
ACE Inhibitor/AT II Antagonists	2994 (30.15)	212564 (22.21)	123 (11.50)	2641 (6.83)	1458 (14.62)	93057 (10.38)	8696 (33.81)	659644 (26.14)	4870 (25.17)	339351 (18.44)	2598 (19.23)	165092 (13.39)
Low-dose Aspirin	1142 (11.50)	51668 (5.40)	168 (15.70)	2172 (5.61)	2788 (27.95)	150118 (16.74)	7479 (29.08)	483207 (19.15)	4669 (24.13)	279314 (15.18)	4518 (33.44)	251489 (20.40)
Beta Blockers	3334 (33.58)	227145 (23.73)	157 (14.67)	2866 (7.41)	2035 (20.40)	123150 (13.73)	5168 (20.09)	327230 (12.97)	2753 (14.23)	160699 (8.73)	2640 (19.54)	167888 (13.62)
	**GePaRD**		**IPCI**		**PHARMO**		**SISR**		**OSSIFF**		**THIN**	
	**Cases**	**Controls**	**Cases**	**Controls**	**Cases**	**Controls**	**Cases**	**Controls**	**Cases**	**Controls**	**Cases**	**Controls**
	**N = 9,930**	**N = 957,016**	**N = 1,070**	**N = 38,688**	**N = 9,974**	**N = 896,907**	**N = 25,719**	**N = 2,523,118**	**N = 19,349**	**N = 1,840,368**	**N = 13,511**	**N = 1,232,506**
	**N (%)**	**N (%)**	**N (%)**	**N (%)**	**N (%)**	**N (%)**	**N (%)**	**N (%)**	**N (%)**	**N (%)**	**N (%)**	**N (%)**
Calcium Channel Blockers	1783 (17.96)	117954 (12.33)	110 (10.28)	1350 (3.49)	1257 (12.60)	66124 (7.37)	8321 (32.35)	552807 (21.91)	5208 (26.92)	316085 (17.18)	2533 (18.75)	149374 (12.12)
Diuretics	1768 (17.80)	112344 (11.74)	107 (10.00)	2069 (5.35)	1413 (14.17)	91455 (10.20)	5882 (22.87)	422360 (16.74)	2991 (15.46)	196169 (10.66)	3336 (24.69)	226862 (18.41)
Glucocorticoids	590 (5.94)	39520 (4.13)	42 (3.93)	610 (1.58)	636 (6.38)	33545 (3.74)	1467 (5.70)	93448 (3.70)	974 (5.03)	61070 (3.32)	955 (7.07)	51033 (4.14)
Lipid lowering agents	2413 (24.30)	163192 (17.05)	152 (14.21)	3415 (8.83)	2421 (24.27)	161369 (17.99)	5445 (21.17)	366094 (14.51)	3732 (19.29)	232329 (12.62)	4451 (32.94)	277358 (22.50)
Nitrates	1053 (10.60)	28570 (2.99)	65 (6.07)	386 (1.00)	1554 (15.58)	39619 (4.42)	5508 (21.42)	196115 (7.77)	4004 (20.69)	27934 (6.95)	2744 (20.31)	67949 (5.51)
Oral Contraceptives#	2 (0.09)	103 (0.05)	5 (1.28)	171 (1.22)	221 (2.63)	14421 (1.79)	41 (1.19)	2619 (0.84)	24 (0.21)	2051 (0.18)	20 (0.40)	2782 (0.61)
Combinations of Hypertensive Drugs	2086 (21.01)	159257 (16.64)	27 (2.52)	1044 (2.70)	414 (4.15)	31157 (3.47)	5793 (22.52)	514022 (20.37)	3237 (16.73)	273082 (14.84)	160 (1.18)	11411 (0.93)
Platelet Aggregation Inhibitors	560 (5.64)	18102 (1.89)	31 (2.90)	276 (0.71)	486 (4.87)	20184 (2.25)	2531 (9.84)	118333 (4.69)	1468 (7.59)	64830 (3.52)	917 (6.79)	29321 (2.38)
Postmenopausal Hormone Therapy#	225 (10.1)	28504 (13.6)	11 (2.81)	267 (1.90)	161 (1.91)	15628 (1.94)	201 (5.83)	24929 (8.0)	150 (1.29)	19060 (1.66)	328 (6.51)	30551 (6.67)
Potential Confounders***												
Alcohol Abuse	208 (2.09)	13603 (1.42)	75 (7.01)	2803 (7.25)	15 (0.15)	1284 (0.14)	27 (0.10)	3259 (0.13)	32 (0.17)	2746 (0.15)	1150 (8.51)	102040 (8.28)
Atrial Fibrillation and Flutter	563 (5.67)	43774 (4.57)	51 (4.77)	458 (1.18)	38 (0.38)	2810 (0.31)	401 (1.56)	26752 (1.06)	248 (1.28)	15609 (0.85)	85 (0.63)	5045 (0.41)
Chronic Liver Disease	1118 (11.26)	101200 (10.57)	22 (2.06)	635 (1.64)	5 (0.05)	486 (0.05)	174 (0.68)	13702 (0.54)	98 (0.51)	8435 (0.46)	12 (0.09)	1037 (0.08)
Obesity	1439 (14.49)	102922 (10.75)	17 (1.59)	510 (1.32)	26 (0.26)	1651 (0.18)	162 (0.63)	8405 (0.33)	47 (0.24)	2565 (0.14)	1167 (8.64)	83424 (6.77)
Osteoarthritis	2196 (22.11)	208937 (21.83)	28 (2.62)	1205 (3.11)	122 (1.22)	10137 (1.13)	330 (1.28)	29986 (1.19)	256 (1.32)	20101 (1.09)	1711 (12.66)	130432 (10.58)
	**GePaRD**		**IPCI**		**PHARMO**		**SISR**		**OSSIFF**		**THIN**	
	**Cases**	**Controls**	**Cases**	**Controls**	**Cases**	**Controls**	**Cases**	**Controls**	**Cases**	**Controls**	**Cases**	**Controls**
	**N = 9,930**	**N = 957,016**	**N = 1,070**	**N = 38,688**	**N = 9,974**	**N = 896,907**	**N = 25,719**	**N = 2,523,118**	**N = 19,349**	**N = 1,840,368**	**N = 13,511**	**N = 1,232,506**
	**N (%)**	**N (%)**	**N (%)**	**N (%)**	**N (%)**	**N (%)**	**N (%)**	**N (%)**	**N (%)**	**N (%)**	**N (%)**	**N (%)**
Other Cardiovascular Disease	1990 (20.04)	155371 (16.23)	73 (6.82)	1420 (3.67)	123 (1.23)	10154 (1.13)	1627 (6.33)	19758 (4.75)	932 (4.82)	64362 (3.50)	258 (1.91)	14793 (1.20)
Peripheral arterial diseases	883 (8.89)	46968 (4.91)	14 (1.31)	306 (0.79)	26 (0.26)	809 (0.09)	408 (1.59)	12054 (0.48)	276 (1.43)	8672 (0.47)	3 (0.02)	134 (0.01)
Rheumatoid Arthritis and Inflammatory Polyarthritis	712 (7.17)	56886 (5.94)	119 (11.12)	3650 (9.43)	122 (1.22)	8498 (0.95)	157 (0.61)	8087 (0.32)	129 (0.67)	8584 (0.47)	1182 (8.75)	82784 (6.72)
Anticoagulants	589 (5.93)	48421 (5.06)	27 (2.52)	486 (1.26)	610 (6.12)	41528 (4.63)	1788 (6.95)	131880 (5.23)	1359 (7.02)	93024 (5.05)	402 (2.98)	29515 (2.39)
Cardiac Glycosides	396 (3.99)	22597 (2.36)	11 (1.03)	126 (0.33)	188 (1.88)	12236 (1.36)	1326 (5.16)	99367 (3.94)	898 (4.64)	60803 (3.30)	264 (1.95)	16973 (1.38)
CYP2C9 Inducer drugs†	2 (0.02)	80 (0.01)			1 (0.01)	129 (0.01)					2 (0.01)	232 (0.02)
CYP2C9 Inhibitor drugs‡	107 (1.08)	7920 (0.83)	10 (0.93)	94 (0.24)	128 (1.28)	8206 (0.91)	868 (3.37)	60204 (2.39)	636 (3.29)	43783 (2.38)	143 (1.06)	7675 (0.62)

*Age and sex are matching criteria.

# Percentage only in females.

† Includes Carbamazepine, Norethisterone (and estrogen combination) and Prednisone.

‡ Includes Cimetidine, Omeprazole, Pantoprazole, Lansoprazole, Rabeprazole, Ticlopidine, Indometacin, Probenecid, Oxcarbazepine, Felbamate, Topiramate, Fluoxetine, Fluvoxamine, Modafinil and Ketoconazole.

** Confounders including diseases and smoking were assessed at 12 months prior to cohort entry.

*** Confounders including use of drugs were assessed at 30 or 90 days before indexdate.

**Table 2:** Association between current use of an individual NSAID and risk of AMI compared with past use of any NSAID pooled by meta-analysis approach (random and fixed effects) and by unweighted (matched set) pooled dataset.

			Meta-analysis approach (random effects)			Meta-analysis (fixed effects)	Pooled dataset
	Cases N	Controls N	Number of databases	ORmeta (95% CI)	I^2^ *	ORfixed (95% CI)	ORpooled (95% CI)
**Past Use of any NSAID**	55,657	5,307,077	6	1 (ref)		1 (ref)	1 (ref)
**Recent use of any NSAID**	23,896	2,181,526	6	1.08 (1.04 to 1.11)	65.27	1.08 (1.06 to 1.09)	1.08 (1.06 to 1.11)
**Current use of:**							
Aceclofenac	214	20,370	4	1.04 (0.90 to 1.19)	0.00	1.04 (0.9 to 1.19)	1.08 (0.85 to 1.36)
Acemetacin	14	1,178	1				1.00 (0.58 to 1.71)
Celecoxib	886	76,132	5	1.15 (0.91 to 1.46)	66.61	1.12 (1.05 to 1.20)	1.15 (1.05 to 1.25)
Dexibuprofen	41	2,651	2	1.15 (0.79 to 1.68)	0.00	1.15 (0.79 to 1.68)	1.06 (0.61 to 1.82)
Dexketoprofen	9	723	1				1.01 (0.50 to 2.04)
Diclofenac	3,064	230,213	6	1.31 (1.17 to 1.48)	60.03	1.32 (1.27 to 1.37)	1.28 (1.22 to 1.34)
Diclofenac, combinations	399	27,923	6	1.27 (1.11 to 1.44)	18.76	1.27 (1.15 to 1.40)	1.30 (1.17 to 1.45)
Etodolac	37	2,761	1				1.07 (0.76 to 1.50)
Etoricoxib	497	37,478	6	1.28 (1.17 to 1.40)	11.71	1.27 (1.16 to 1.39)	1.39 (1.24 to 1.57)
Flurbiprofen	27	1,972	2	1.05 (0.66 to 1.67)	0.00	1.05 (0.66 to 1.67)	1.00 (0.56 to 1.78)
Ibuprofen	1,564	119,219	6	1.24 (1.04 to 1.48)	61.38	1.25 (1.19 to 1.32)	1.25 (1.18 to 1.33)
Indometacin	196	11,789	5	1.47 (1.27 to 1.70)	0.00	1.47 (1.27 to 1.70)	1.51 (1.28 to 1.80)
Ketoprofen	559	47,969	3	1.12 (1.03 to 1.22)	0.00	1.12 (1.03 to 1.22)	1.00 (0.86 to 1.16)
Ketorolac	272	11,732	2	2.06 (1.83 to 2.32)	0.00	2.06 (1.83 to 2.32)	1.80 (1.49 to 2.18)
Lornoxicam	40	3,095	2	1.08 (0.77 to 1.51)	0.00	1.08 (0.77 to 1.51)	1.08 (0.62 to 1.87)
Mefenamic acid	12	981	1				1.02 (0.55 to 1.90)
Meloxicam	492	38,806	6	1.18 (1.08 to 1.29)	0.00	1.18 (1.08 to 1.29)	1.13 (1.02 to 1.27)
Nabumetone	46	3,795	4	1.03 (0.76 to 1.40)	0.00	1.03 (0.76 to 1.40)	1.03 (0.72 to 1.47)
Naproxen	486	38,659	6	1.19 (0.95 to 1.49)	47.10	1.18 (1.08 to 1.29)	1.22 (1.10 to 1.35)
Nimesulide	1,652	133,462	2	1.16 (1.11 to 1.22)	0.00	1.16 (1.10 to 1.22)	1.12 (1.03 to 1.22)
Oxaprozin	22	2,709	2	0.93 (0.63 to 1.38)	0.00	0.93 (0.63 to 1.38)	0.97 (0.52 to 1.79)
Piroxicam	636	51,898	5	1.17 (0.99 to 1.37)	34.02	1.20 (1.10 to 1.30)	1.27 (1.13 to 1.42)
Proglumetacin	11	930	1				1.00 (0.41 to 2.47)
Rofecoxib	690	51,674	4	1.26 (1.17 to 1.36)	0.00	1.26 (1.17 to 1.36)	1.30 (1.19 to 1.43)
Sulindac	11	494	1				1.01 (0.48 to 2.15)
Tenoxicam	32	3,104	2	1.02 (0.71 to 1.46)	0.00	1.02 (0.71 to 1.46)	0.99 (0.56 to 1.74)
Tiaprofenic acid	8	710					1.01 (0.49 to 2.10)
Valdecoxib	25	2,159	3	1.00 (0.66 to 1.52)	0.00	1.00 (0.66 to 1.52)	1.07 (0.58 to 1.99)

* A high level of heterogeneity is present with an I^2^ value above 75%.

**Fig 1:**
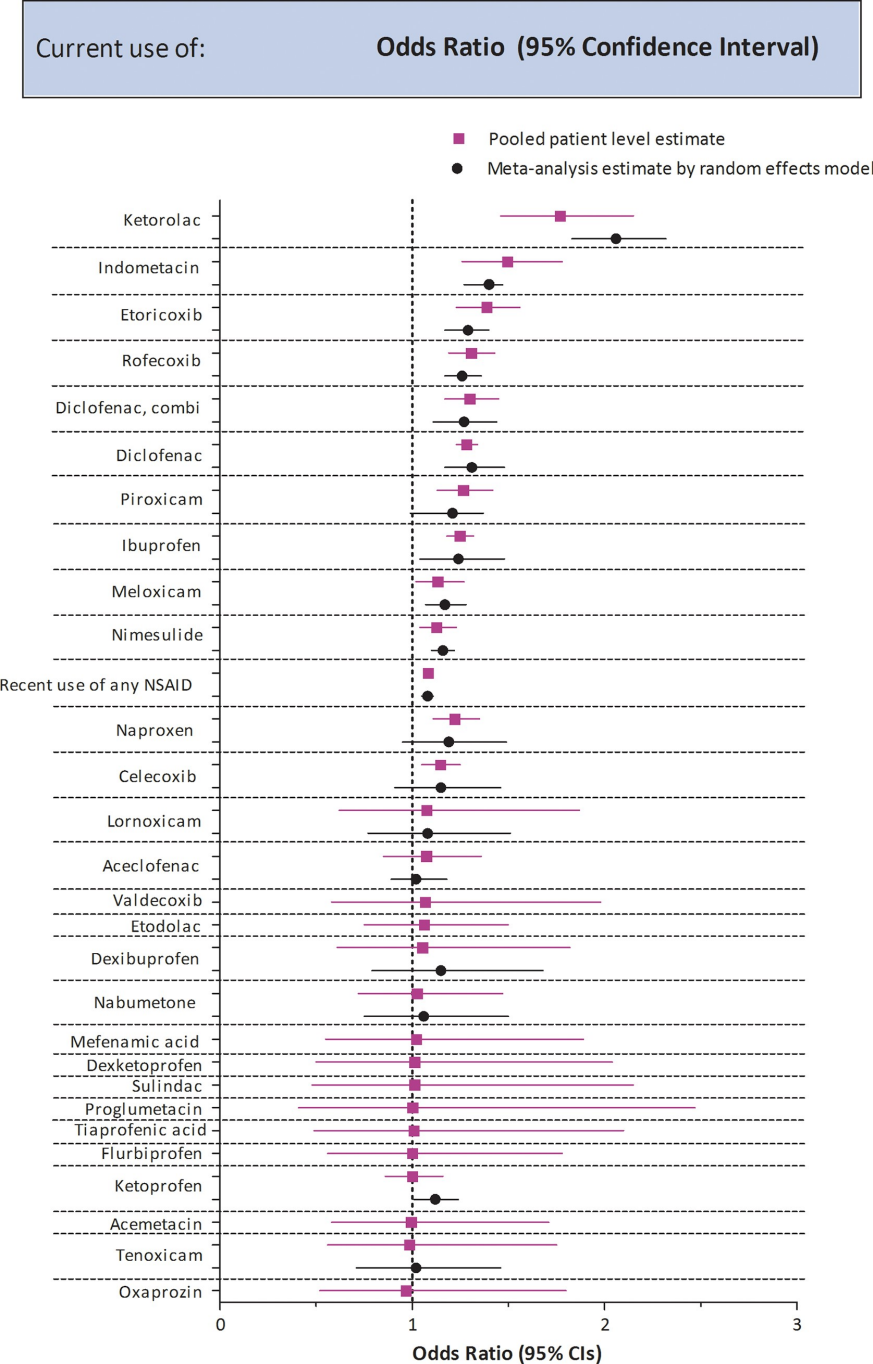
Adjusted risk estimates of AMI for current use of individual NSAIDs versus past use of any NSAID in the pooled analysis plotted in descending order from the NSAID with highest point estimate without taking the confidence limits into account 1) meta-analytic pooling by random effects model and 2) on individual datasets.

Whereas UK, NL and Germany use similar NSAIDS, Italy uses quite a different range of NSAIDs, for 11 individual NSAIDs data were only available from Italy (S5 Table) Meta-analytic estimates of the adjusted ORs across databases could be calculated for 21 NSAIDs. The adjusted OR for current use ranged between 0.93 for oxaprozin to 2.06 for ketorolac (Fig 1, Table 2), but the width of the confidence intervals vary. Ten NSAIDs were associated with a statistically significantly increased risk. For 11 NSAIDs we did not observe a significant increased association with AMI in the meta-analytic pooling approach for these NSAIDs the upper 95% limits was below two. For most NSAIDs no heterogeneity was seen according to the estimated I^2^ across databases (Table 2).

The one stage pooling (Fig 1, Table 2), yielded estimates for 28 individual NSAIDs, for most NSAIDs the two and one stage pooled estimates were quite similar. Results of this combined analysis showed that the risk of AMI is statistically significantly elevated for 12 NSAIDs. Compared to past use of any NSAID the OR was highest for ketorolac, followed by indometacin, etoricoxib and rofecoxib (Fig 1), though the 95% confidence limits overlap between these NSAIDs. For 16 NSAIDs the association with AMI was not statistically significantly elevated, but for four of those NSAIDs the 95%CI upper limits were above two. The relationship between COX-2 potency and the risk of AMI for normal daily doses (PDD/DDD 0.8–1.2) is seen in S2 Fig.

**Fig 2:**
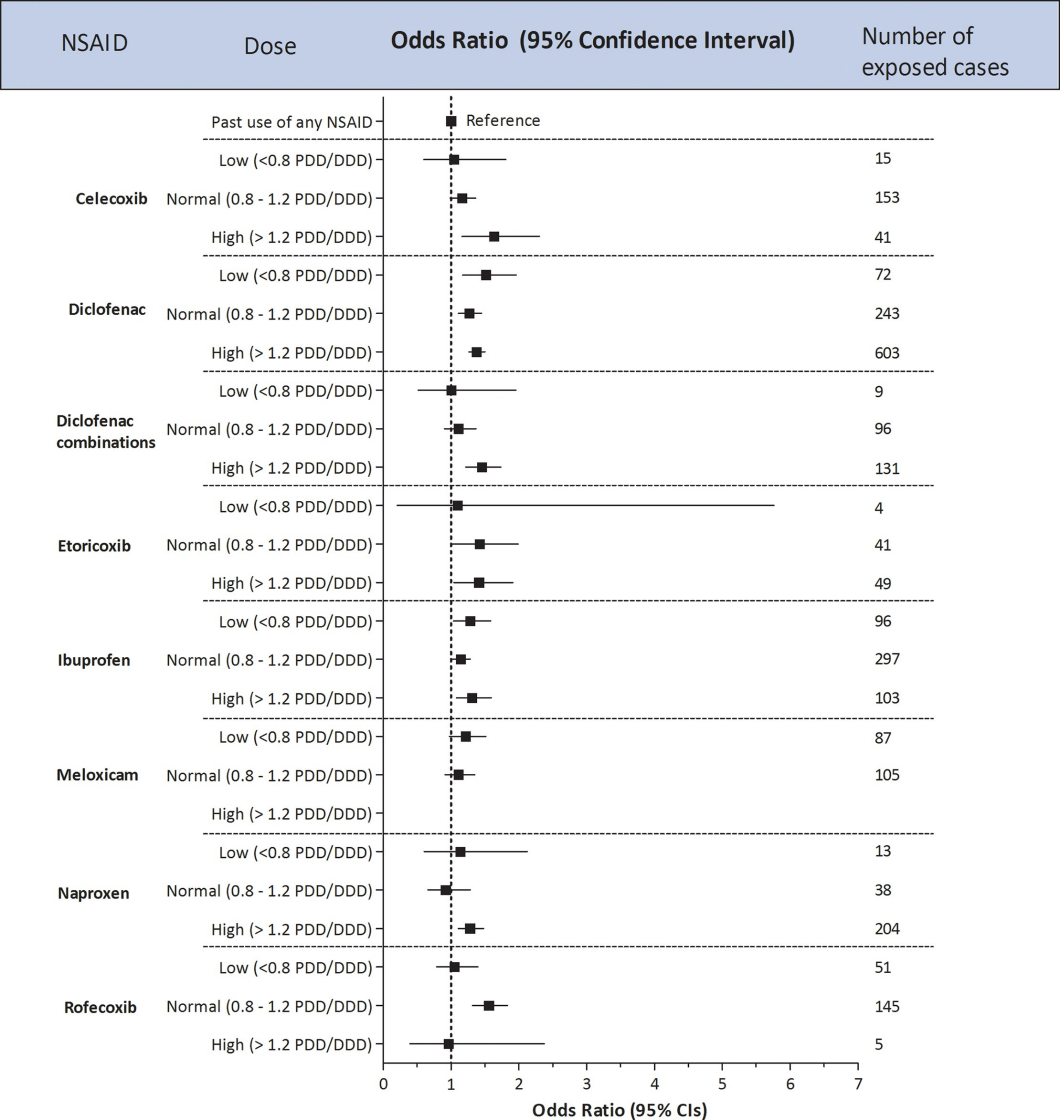
Adjusted risk estimates for AMI in current users for dose of use of individual NSAIDs in three databases pooled (THIN, IPCI, PHARMO), using past use of any NSAID as common reference group. PDD, prescribed daily dose; DDD, defined daily dose. Number of exposed cases do not add up to all current users of that particular NSAID in all three databases pooled as dose information could have been missing.

Categorical dose response analyses versus past use of any NSAID in the subset of databases with prescription regimens (THIN, IPCI, PHARMO) showed that higher doses of celecoxib, the fixed combination of diclofenac and misoprostol, etoricoxib and naproxen increased the risk of AMI (Fig 2). Continuous dose-response curves with cubic splines showed a significant dose response for diclofenac only (S3 Fig). For duration, no clear patterns were seen (S4 Fig). The risk of AMI seemed highest with shortest duration for diclofenac, which was also seen for ibuprofen and rofecoxib although confidence limits between categories overlapped.

Stratification by AMI risk factors for each NSAID did not reveal consistent patterns for potential effect modifiers across individual NSAIDs (S7 and S9 Tables).

## Discussion

In this unique multinational case-control study nested in a new user NSAID cohort of more than 8.5 million persons, we assessed the association with AMI for 28 individual NSAIDs. Following rofecoxib’s withdrawal many single studies have been conducted using different protocols and definitions.[4,11,37–39] Meta-analyses of these studies identified large variability between studies and several methodological issues such as lack of information on dose effects, and lack of estimates for less frequently used NSAIDs.[12,14,40] The SOS study is a major leap forward from those regular meta-analyses of different studies because it combines patient-level data that have been collected using a common data model, protocol, definitions, data transformation and analysis.

The highest risk of AMI was observed for current use of ketorolac. Various other widely used nsNSAIDs, such as indometacin, diclofenac, piroxicam, ibuprofen, naproxen, meloxicam and nimesulide; and selective COX-2 inhibitors, such as etoricoxib, rofecoxib and celecoxib, were associated with a small increase in risk of AMI.

Our risk estimates are lower (except for naproxen and etoricoxib) than the estimates from meta-analyses of clinical trials[13] for six NSAIDs (celecoxib, diclofenac, etoricoxib, ibuprofen, naproxen, rofecoxib) that have been studied in RCT meta-analyses. Explanation might be that some of the large efficacy trials were done for comparison of gastro-intestinal effects, with higher dosages than in everyday practice.[41] For nine NSAIDs for which we have evidence from meta-analyses of observational studies the SOS estimates were within the width of the CIs, providing reassurance about consistency.[12,13] Also, estimates are in line with an individual patient data meta-analysis for celecoxib, rofecoxib, diclofenac, ibuprofen and naproxen.[14]

What are the key findings for individual nsNSAIDs in this study that are new and relevant for clinicians? First, diclofenac (median dose 150 mg, 1.5 DDD) is very frequently used and associated with AMI in a similar magnitude as rofecoxib (median dose 25 mg, 1 DDD). Recent studies confirm this. [12–14] With support of SOS data, EMA had decided in 2013 to restrict the use of diclofenac.[42] Second, there was one nsNSAID with even higher relative risk of AMI: ketorolac, which was only used in Italy. The third finding is that we observed a 22% increase in risk of AMI with naproxen use, which is in contrast with some previous studies[11–13,37,43,44] but in line with others.[11,14,43,45–47] Prior studies showed an increase in risk between 14%[46]-19%[47] for naproxen as compared to remote NSAID use, a similar comparator group we used. Although trials provide higher level evidence for the specific study population, our finding on naproxen is in line with some clinical trials.[48,49] In the TARGET trial no significant differences were observed between the different NSAIDs and the incidence of AMI.[49] Additionally in patients free of CV disease, rate of CV disease was similar for celecoxib as with nsNSAIDs.[50] A recent meta-analysis of individual patient data investigated only five NSAIDs (celecoxib, rofecoxib, ibuprofen, diclofenac and naproxen) and showed that compared to no use of NSAIDs in the previous year, the adjusted ORs were in line with our results (Table 3).[14] Also, it demonstrated that the risk of AMI for celecoxib was comparable to that of nsNSAIDs including naproxen. However, in the meta-analysis they used as reference group no use of NSAID in the previous year, which explains the higher risks observed than in our study.[14] Additionally, our findings regarding naproxen may be explained by differences in dosing. In the dose response analyses that included UK and Dutch databases, the OR was only statistically significantly increased for the highest dose of naproxen as compared to past use of any NSAID and the median dose for naproxen was 2 times the daily recommended dose, whereas other NSAIDs were prescribed lower doses (S4 Table).

**Table 3:** Risk estimates of AMI for individual NSAIDs from current SOS study, meta-analysis from observational studies[12] and randomized clinical trials[13], using major vascular events as outcome.

	SOS study (pooled dataset)	Meta-analysis published observational studies	Composite Endpoint from meta-analysis of randomized clinical trials#	Meta-analysis individual patient data†
	Adjusted ORpooled (95% CI)	Relative Risk (random effects)	Adjusted Rate Ratio	Adjusted odds ratio
Reference group	Past Use of any NSAID	No or remote NSAID use	Placebo	No use of NSAID in previous year
**Current use of:**				
Aceclofenac	1.08 (0.85 to 1.36)			
Acemetacin	1.00 (0.58 to 1.71)			
Celecoxib	1.15 (1.05 to 1.25)	1.23 (1.00 to 1.52)*	1.36 (0.91−2.02)	1.24 (0.91–1.82)
Dexibuprofen	1.06 (0.61 to 1.82)			
Dexketoprofen	1.01 (0.50 to 2.04)			
Diclofenac	1.28 (1.22 to 1.34)	1.41 (1.08 to 1.86)*	1.41 (1.12 to 1.78)	1.50 (1.06–2.04)
Diclofenac, combinations	1.30 (1.17 to 1.45)			
Etodolac	1.07 (0.76 to 1.50)	1.55 (1.16 to 2.06)		
Etoricoxib	1.39 (1.24 to 1.57)	1.97 (1.35 to 2.89)	0.83 (0.18−3.77)	
Flurbiprofen	1.00 (0.56 to 1.78)			
Ibuprofen	1.25 (1.18 to 1.33)	1.20 (0.97 to 1.48)*	1.44 (0.89−2.33)	1.48 (1.00–2.26)
Indometacin	1.51 (1.28 to 1.80)	1.40 (1.21 to 1.62)		
Ketoprofen	1.00 (0.86 to 1.16)			
Ketorolac	1.80 (1.49 to 2.18)			
Lornoxicam	1.08 (0.62 to 1.87)			
Mefenamic acid	1.02 (0.55 to 1.90)			
Meloxicam	1.13 (1.02 to 1.27)	1.25 (1.04 to 1.49)		
Nabumetone	1.03 (0.72 to 1.47)			
Naproxen	1.22 (1.10 to 1.35)	0.85 (0.73 to 1.00)*	0.93 (0.69−1.27)	1.53 (1.07–2.33)
Nimesulide	1.12 (1.03 to 1.22)			
Oxaprozin	0.97 (0.52 to 1.79)			
Piroxicam	1.27 (1.13 to 1.42)			
Proglumetacin	1.00 (0.41 to 2.47)			
Rofecoxib	1.30 (1.19 to 1.43)	1.43 (1.21 to 1.66)*	1.38 (0.99−1.94)	1.58 (1.07–2.17)
Sulindac	1.01 (0.48 to 2.15)			
Tenoxicam	0.99 (0.56 to 1.74)			
Tiaprofenic acid	1.01 (0.49 to 2.10)			
Valdecoxib	1.07 (0.58 to 1.99)			

* in new users exposed to NSAIDs

# The outcome major vascular events included non-fatal MI, coronary death, MI or CHD death, non-fatal stroke, stroke death, any stroke and other vascular death). Daily dose studied in Clinical Trials: Diclofenac (150 mg); Ibuprofen (2400 mg); Naproxen (1000 mg); Celecoxib (100–800 mg, typical doses contributing the majority of information on major vascular events 400 mg); Rofecoxib (12.5–125 mg; typical dose 25 mg); Lumiracoxib (100–800 mg; typical dose 200 mg); Etoricoxib (5–120 mg; typical dose 60/90 mg); Valdecoxib (1–80 mg; typical dose 20 mg)

† current use is classified as use of any dose for 1–7 days

The key findings related to coxibs are that the relative risk of AMI for etoricoxib was higher than for diclofenac and almost equal to that for rofecoxib, though the CIs were overlapping. An increased risk for etoricoxib was also found in the meta-analysis of observational studies.[12] Although etoricoxib has not been studied extensively in placebo-controlled trials, effects of etoricoxib, rofecoxib and celecoxib seemed similar in trials comparing COX-2 selective inhibitors to diclofenac.[13] In the MEDAL trial the rate of thrombotic cardiovascular events was similar between the diclofenac and the etoricoxib group (HR 0.95; 95%CI:0.81–1.11), confirming our findings.[51] Our results regarding celecoxib are consistent with the meta-analysis of observational studies and show only a very small increase in risk.[12] Also, a recent published trial showed that celecoxib is noninferior to ibuprofen and naproxen for cardiovascular safety.[48]

Dose response analyses showed that AMI risk varied by dose, which means that the overall estimates are largely driven by the dose that will be used in a country. Lower doses generally, but not for all drugs, have a lower risk in the databases we could use to study this. When plotting the potency of individual NSAIDs in the degree of COX-2 inhibition by normal therapeutic doses, it may seem to show a correlation in line with a previous study but we could only look at 6 different NSAIDs as we needed daily dose information which was available in THIN, IPCI and PHARMO.[4] This supports the previously suggested hypothesis that the extent of inhibition of COX-2–dependent prostacyclin may represent an independent determinant of the increased risk of nonfunctional suppression of platelet COX-1.[5] However, as the degree of relative COX-1 and COX-2 inhibition varies between NSAIDs and between the non-selective and selective COX-2 inhibitors one may expect this suppression for all NSAIDs.[4]

Stratification for concurrent use of aspirin, lipid lowering drugs, presence of ischemic heart disease, sex, and age several significant interactions were observed with isolated NSAIDs. E.g. the risk for AMI associated with diclofenac was higher in females than males. The risk was statistically significantly higher in younger persons (<60 years) than older for naproxen. Our findings are consistent with published findings showing that the relative risk of AMI in current users of NSAIDs is higher in low-CV risk subjects, than in high-risk subjects. However, it should be considered that the absolute risk is much higher in high-CV risk patients due to higher background rates. [11–13,43]

Strength of the current study is the common distributed approach for collaboration[28] which allowed us to analyze the data on one stage and two stage pooling being able to more stratified and detailed analysis in one stage pooling while preserving differences in confounder availability by two stage pooling.[52] Another method to summarize individual-level data from a large number of covariates by calculating a propensity score, however although it allows for consistency of adjustment for common confounders across the data sites, it does not fully use additional confounder information that may be available at certain data sites. The final obtained exposure estimates in each of the data sites should still be summarized in a summary and overall statistic.[53] We acknowledge the following limitations. Since NSAID use was assessed through computerized prescriptions/dispensing by physicians, over-the-counter NSAID use was not captured. Channeling of COX-2 inhibitors to high GI-risk patients might have occurred and was addressed by matching on calendar time beyond age, sex and database. Additionally, we adjusted for a large range of known risk factors for AMI. The matched and adjusted estimates were very similar, indicating that most of the potential confounding variables were time, sex and age-related and taken care of by the matching. Moreover, we stratified by calendar time and saw no significant difference, also the prevalence of risk factors did not change before and after 2004. Restriction of the population to patients without risk factors (where no channeling would occur) did not change the conclusions (S9 Table), although any remaining residual confounding or confounding by indication may be present and resulted in biasing the results. Although dose analysis was performed to verify dose-response relationships for NSAIDs and for comparison across NSAIDs independent of dosage used, we only had dose available in three databases (THIN, IPCI, PHARMO) which is a limitation of our study. We were not able to match all potential AMI cases to controls in the new NSAID user cohort, due to the different matching criteria that were applied to limit confounding by calendar time, age, sex and database site, this improves internal validity but potentially limits external validity, most likely in patients at extreme age ranges.

Concluding, this study provides risk estimates for the association between the use of 28 different NSAIDs and the risk of AMI. Evaluating the variability of AMI risk across these NSAIDs in real life practice circumstances showed that for twelve individual NSAIDs the risk of AMI is significantly increased and sixteen NSAIDs there was no significantly increased risk. Although COX-2 potency is correlated with risk, it is not a strong feature determining the cardiovascular safety of NSAIDs as was initially advocated. This should warrant doctors and pharmacists when prescribing or dispensing NSAIDs to patients at CV risk.

## Supporting information

S1 Checklist Strobe checklist. (DOC)

S1 Table Characteristics of participating databases. (DOCX)

S2 Table NSAIDs included in the SOS project with annual prevalence of users per 100,000 person-years. (DOCX)

S3 Table List of diagnostic codes (plus related label) for acute myocardial infarction case identification in electronic medical records stratified by coding system. (DOCX)

S4 Table Doses considered in the current study in the three databases that captured prescribed doses (THIN, IPCI, PHARMO). (DOCX)

S5 Table Association between current use of individual NSAIDs and the risk of AMI compared with past use of any NSAID in individual databases. (DOCX)

S6 Table Characteristics of NSAID users by NSAID. (DOCX)

S7 Table Effect modification by important (proxy) risk factors for AMI on the matched pooled dataset. (DOCX)

S8 Table NSAIDs included in the SOS project with mean duration of use in days per person. (DOCX)

S9 Table Association between current use of individual NSAIDs and the risk of AMI compared with past use of any NSAID before index date in individual databases in patients with a low cardiovascular risk profile. (DOCX)

S1 Fig Flowchart of source population and study population per database. (DOCX)

S2 Fig Relation between degree of inhibition of whole blood COX-2 and risk of AMI for individual NSAIDs in the three databases that included doses (THIN, IPCI, PHARMO). (DOCX)

S3 Fig Dose response estimated by fractional polynomial regression for diclofenac. (DOCX)

S4 Fig Adjusted risk estimates of AMI in current users of individual NSAIDs for duration of use in three databases pooled (THIN, IPCI, PHARMO) using short duration (7–29 days) as reference group. (DOCX)

## Abbreviations

AMI: Acute Myocardial Infarction
BNF: British National Formulary
COX: Cyclo-oxygenase
Coxibs: Selective COX-2 inhibitors
CV: Cardiovascular
DDD: Defined daily dose
EMA: European Medicines Agency
EU: European Union
GE: Germany
GePaRD: German Pharmacoepidemiological Research Database
ICD-9 CM: International Classification of Diseases, 9^th^ revision, Clinical Modification
ICD-10: International Classification of Diseases, 10^th^ Revision
ICPC: International Classification of Primary Care
IPCI: Integrated Primary Care Information Project
IT: Italy
NL: the Netherlands
NSAID: Non-steroidal anti-inflammatory drug
nsNSAID: Non-selective non-steroidal anti-inflammatory drug
OSSIFF: Osservatorio Interaziendale per la Farmacoepidemiologia e la Farmacoeconomia (Network of Local Health Autorities for Pharmacoepidemiology and Pharmaceconomics)
OR: Odds ratio
OTC: Over-the-counter
SISR: Sistema Informativo Sanitario Regionale (Regional Health Information System)
THIN: The Health Improvement Network
UK: United Kingdom
WHO: World Health Organization of the United Nations
