# Supplementary material for: Risk of acute myocardial infarction during use of individual NSAIDs: A nested case-control study from the SOS project
Source: PLoS One. 2018 Nov 1;13(11):e0204746. doi: 10.1371/journal.pone.0204746 (PMC6211656; doi:10.1371/journal.pone.0204746)
Supplement: S1 Checklist — (DOC) [file pone.0204746.s001.doc]

**S1 Checklist**

STROBE Statement—Checklist of items that should be included in reports of ***case-control studies***

|  | Item No | Recommendation |
| --- | --- | --- |
| **Title and abstract** | 1 | (*a*) Indicate the study’s design with a commonly used term in the title or the abstract  Included in the abstract. |
|  |  | (*b*) Provide in the abstract an informative and balanced summary of what was done and what was found  Included in the abstract. We found in a nested case-control study in naive NSAID users from 6 European databases that the risk of acute myocardial infarction (AMI) was increased for current use of several individual NSAIDs. The risk was highest for ketorolac, but was increased also for several other selective COX-2 and non-selective NSAIDs and was higher when using higher daily doses. The increased risk of AMI should not be considered an effect of some selective COX-2 inhibitors only. |
| Introduction | | |
| Background/rationale | 2 | Explain the scientific background and rationale for the investigation being reported  Non-steroidal anti-inflammatory drugs (NSAIDs) are widely used to reduce inflammation and provide pain relief. They act via reversible, competitive inhibition of cyclo-oxygenase (COX) enzymes. As inhibition of the COX-1 enzyme decreases the production of prostaglandins, gastrointestinal adverse events including ulcerations and bleeding occur often during NSAID use. This led to development of selective COX-2 inhibitors. However, after successful market introduction of selective COX-2 inhibitors concerns were raised about their cardiovascular safety resulting in the voluntary withdrawal of rofecoxib in 2004. Reviews by the United States Food and Drug Administration (FDA) and the European Medicines Agency (EMA) concluded that selective COX-2 inhibitors increase the risk of CV events. It was recommended to avoid selective COX-2 inhibitors in patients with ischemic heart disease, stroke or peripheral arterial disease. At that point in time little information was available about the CV risk of the traditional non-selective (ns) NSAIDs, but further studies showed signals of increased arterial thrombosis risk, particularly when used in high doses and for long-term. Based on the uncertainty, EMA requested a review of the CV safety of nsNSAIDs as well. The Safety of Non-Steroidal Anti-inflammatory Drugs (SOS) project was developed as a research and development project funded by the Directorate General of Research and Innovation of the European Commission under the Seventh Framework Programme to support EMA in their regulatory decision making. This SOS study aimed to assess and compare the risk of AMI associated with the use of individual NSAIDs. |
| Objectives | 3 | State specific objectives, including any prespecified hypotheses  The Safety of Non-Steroidal Anti-inflammatory Drugs (SOS) project is a research and development project funded by the Directorate General of Research and Innovation of the European Commission under the Seventh Framework Programme. Within the SOS project we conducted a nested case-control study to assess and compare the risk of AMI associated with the use of individual NSAIDs in each of the six electronic health care databases. Collaboration between multiple databases from different countries that worked with a common protocol, common definitions for outcome, confounders and exposures, and common data elaboration scripts enabled us to study a wide variety of individual NSAIDs with a large number of exposed cases, resulting in the ability to provide stable relative risk estimates for infrequently used NSAIDs. |
| Methods | | |
| Study design | 4 | Present key elements of study design early in the paper  Nested case-control study in a new users NSAID cohort in 6 European databases, from 4 European countries. This is presented early in the Methods section. |
| Setting | 5 | Describe the setting, locations, and relevant dates, including periods of recruitment, exposure, follow-up, and data collection  European population-based primary care and administrative databases: from Gernany: German Pharmacoepidemiological Research Database (GePaRD); from the United Kingdom: The Health Improvement Network (THIN); the Netherlands: the Integrated Primary Care Information (IPCI) database and PHARMO; from Italy: Osservatorio Interaziendale per la Farmacoepidemiologia e la Farmaeconomia (OSSIFF) and Sistema Informativo Sanitario Regional (SISR). Study periods differed based on data availability between 1999-2011. Data was collected by electronic search for diagnoses and drug exposure. Data on drug exposure included drug prescriptions and dispensings. |
| Participants | 6 | (*a*) Give the eligibility criteria, and the sources and methods of case ascertainment and control selection. Give the rationale for the choice of cases and controls  In each database, we identified a cohort of patients aged ≥18 years who received at least one NSAID prescription (Table S2) during the database-specific study period which started 1 January 1999 or later (Table S1).  The date of first NSAID prescription/dispensing during the study period was defined as cohort entry date, patients were excluded if they received any NSAID prescription in the year before in order to construct a new user cohort and avoid prevalent user bias. Patients needed to have at least one year of continuous database history, to allow uniform assessment of potential confounding factors and exclusion criteria. All subjects with a cancer diagnosis (except non-melanoma skin cancer) during the one year preceding cohort entry were excluded from the cohort. All NSAID cohort members were followed from the date of cohort entry until the date of acute myocardial infarction diagnosis, cancer, death, last data supply, transferring out of the database, or end of the study period, whichever was earliest.  The outcome was a first hospitalization with a discharge diagnosis code of acute myocardial infarction (AMI) (GePaRD, PHARMO, OSSIFF, and SISR) or a first diagnosis of an AMI (THIN and IPCI) during follow-up (Table S3). The date of recorded diagnosis or admission date of AMI was used as index date. Within each database, up to 100 controls were matched to each case by risk set sampling on age at index date (± 1 year), sex, index date and cohort entry (± 28 days). |
|  |  | (*b*) For matched studies, give matching criteria and the number of controls per case  Within each database, a total of 100 controls per each case were randomly selected from the study cohort and matched on age at index date (± 1 year), sex, index-date and follow-up time in cohort (± 28 days).  Overall, a total of 7,488,603 controls were matched to cases with a median of 99 controls per case ranging from 24 in IPCI to 100 controls per case in GePaRD |
| Variables | 7 | Clearly define all outcomes, exposures, predictors, potential confounders, and effect modifiers. Give diagnostic criteria, if applicable  An AMI case was defined as a patient with an acute myocardial infarction (Supplementary Table 3 for ICD-9, ICD-10, READ and ICPC codes included). The date of diagnosis of the AMI was used as the index date.  NSAID prescription (ATC codes (or equivalent BNF code): M01AA to M01AX, excluding M01AX05, M01AX12, M01AX14, M01AX21, M01AX24, M01AX25, and M01AX26) during the database-specific study period which started at the first of January 1999 or later, depending on data availability.  The date of first NSAID prescription or dispensing during the study period was defined as the date of cohort entry. Subjects receiving any NSAID prescription during the one year prior to cohort-entry were excluded in order to construct a new user cohort to avoid potential biases derived from the inclusion of prevalent users. Patients were required to have at least one year of continuous DB history, to allow uniform assessment of potential confounding factors and exclusion criterion.  Exposure to individual NSAIDs was obtained from either prescriptions (THIN and IPCI) or from outpatient drug dispensings claims (GePaRD, PHARMO, OSSIFF, and SISR). Duration of a single NSAID dispensing/prescription was obtained by dividing the total units by the daily number of units prescribed (THIN, IPCI, and PHARMO: prescribed duration), for other databases standard durations were used based on the country specific defined daily dose (DDD) values.  Classification of recency of exposure to individual NSAIDs was based on the interval between index date and the end of the most recent NSAID use before the index date. If the exposure period 1) overlapped or ended within 14 days before index date NSAID use was classified as ‘current’ use; 2) ended between 15 and 183 days before the index date as ‘recent’ use and; 3) ended 184 days or more before the index date as ‘past’ use. Duration of current use was then classified into very short (1-6 days), short (7-29), medium (30-89) and long (≥ 90). If multiple NSAIDs were used in the current period, NSAID use was distributed to current use of all NSAIDs. Current use of an NSAID always overruled past use of other NSAIDs if patients switched between NSAIDs.  In IPCI, THIN and PHARMO the daily dose of NSAID was estimated from the prescribing regimen and strength. Dose of current exposure to each individual NSAID was classified using the ratio of prescribed daily dose compared to DDD. For categorical analysis dose categories were defined as low dose (<0.8 DDD), normal dose (0.8-1.2 DDD) and high dose (≥1.3 DDD).  Covariates were classified into a-priori risk factors (history of ischemic heart disease (excluding AMI); history of stroke; heart failure; diabetes mellitus type 2; hyperlipidemia; smoking; use of ACE inhibitors, antithrombotic agents, low-dose aspirin, beta blockers, calcium channel blockers, diuretics, glucocorticoids, nitrates, oral contraceptives, platelet aggregation inhibitors, lipid lowering drugs and postmenopausal hormone therapy) or potential confounders (obesity, osteoarthritis and use of anticoagulants). They were measured at 12 months prior to cohort entry or at 30 or 90 days before index date. |
| Data sources/ measurement | 8* | For each variable of interest, give sources of data and details of methods of assessment (measurement). Describe comparability of assessment methods if there is more than one group  Both for cases and controls we measured variables of interest in the same manner. Presence of risk factor and confounders were determined by electronic searched in available data. Covariates were classified into a-priori risk factors (history of ischemic heart disease (excluding AMI); history of stroke; heart failure; diabetes mellitus type 2; hyperlipidemia; smoking; use of ACE inhibitors, antithrombotic agents, low-dose aspirin, beta blockers, calcium channel blockers, diuretics, glucocorticoids, nitrates, oral contraceptives, platelet aggregation inhibitors, lipid lowering drugs and postmenopausal hormone therapy) or potential confounders (obesity, osteoarthritis and use of anticoagulants). They were measured at 12 months prior to cohort entry or at 30 or 90 days before index date. |
| Bias | 9 | Describe any efforts to address potential sources of bias  By using a new user design underascertainment of AMI events at start of NSAID therapy and the inability to control for risk factors that may be modified by NSAIDs were mitigated. Second this approach deals with prevalent user bias.  We assessed the magnitude of residual/unmeasured confounding needed to explain the observed results, which showed that very strong confounding still should be present in order to explain the observed findings.  We mitigated against confounding by indication by applying past use of NSAIDs as comparator instead of using ‘no use of NSAIDs’ as reference category. |
| Study size | 10 | Explain how the study size was arrived at  We provide a flow chart in the manuscript and provide the number of study participants early in the results section. |
| Quantitative variables | 11 | Explain how quantitative variables were handled in the analyses. If applicable, describe which groupings were chosen and why.  Not applicable. |
| Statistical methods | 12 | (*a*) Describe all statistical methods, including those used to control for confounding  Baseline characteristics of cases and controls are described by database. To estimate the risk for AMI among current use of an individual NSAID in comparison to past use of any NSAID, matched odds ratios (ORmatched) and matched adjusted odds ratios (ORadj) with 95% Confidence Intervals (CIs) were calculated using conditional logistic regression analyses for each database separately. If five or more exposed cases per database were available, an association measure was calculated. Pooled NSAID-specific ORs (ORmeta) were obtained by a random effects meta-analysis method to account for heterogeneity across databases.[23](#_ENREF_23) Statistical heterogeneity across databases was tested by using a Cochran’s Q statistic and the degree of heterogeneity by I2.  Additionally, pooling of data across databases was performed by combining the matched case control sets without weighting and using a conditional logistic regression adjusted for covariates. This approach has most power and provides one overall risk measure (ORpooled) for all NSAIDs with at least five exposed cases across DBs.  A stepwise approach was used for confounder selection in both approaches: 1) a-priori selected confounders were always included; 2) univariate analyses for each potential confounder with a prevalence of 5% in controls, which were added to the model if Wald p-value was <0.05; 3) backward selection of potential confounders (p-value>0.05).  Categorical duration analyses were performed within current users of each individual NSAID, using short duration (7-29 days) as reference group. Dose analyses were done by categories comparing dose levels to past use of any NSAID and by continuous analyses through restricted cubic splines (3 knots) and through fractional polynomial regression (maximum of 2 terms) which provides greater flexibility to dose-response curves.[24](#_ENREF_24)  Subsequent analyses evaluated the risk of AMI stratified by sex, age (≤60 or >60 years), prior ischemic heart disease, use of aspirin, or lipid lowering drugs. Multiplicative interaction was tested to identify effect modification by stratifying factors. All analyses were performed using SAS Cary, NC version 9.2. |
|  |  | (*b*) Describe any methods used to examine subgroups and interactions  Subsequent analyses evaluated the risk of AMI stratified by AMI risk factors. Multiplicative interaction was tested to identify effect modification by stratifying factors (use of low-dose aspirin, history of ischemic heart disease). |
|  |  | (*c*) Explain how missing data were addressed |
|  |  | (*d*) If applicable, explain how matching of cases and controls was addressed  Within each database, a total of 100 controls per each case were randomly selected from the study cohort and matched on age at index date (± 1 year), sex, index-date and follow-up time in cohort (± 28 days). |
|  |  | (*e*) Describe any sensitivity analyses |
| Results | | |
| Participants | 13* | (a) Report numbers of individuals at each stage of study—eg numbers potentially eligible, examined for eligibility, confirmed eligible, included in the study, completing follow-up, and analysed  We provide a flow-chart with the number of study participants in each stage of the study. |
|  |  | (b) Give reasons for non-participation at each stage  We provide a flow-chart with the number of study participants in each stage of the study. |
|  |  | (c) Consider use of a flow diagram  We provide a flow-chart with the number of study participants in each stage of the study. |
| Descriptive data | 14* | (a) Give characteristics of study participants (eg demographic, clinical, social) and information on exposures and potential confounders  We provide baseline characteristics of cases and controls in Table 1 by database. |
|  |  | (b) Indicate number of participants with missing data for each variable of interest |
| Outcome data | 15* | Report numbers in each exposure category, or summary measures of exposure  In Table 2 we report the frequency of drug exposure for each individual NSAID by cases and controls. In Table S4 we report the exposure by database. |
| Main results | 16 | (*a*) Give unadjusted estimates and, if applicable, confounder-adjusted estimates and their precision (eg, 95% confidence interval). Make clear which confounders were adjusted for and why they were included  In the results section, Table 2, Table 3, Figure 1, 2 and Table S4 we provide odds ratios, matched and adjusted with their corresponding 95% confidence intervals. |
|  |  | (*b*) Report category boundaries when continuous variables were categorized  Dose and duration-categories for drug exposure is reported in the methods section and in the corresponding tables. |
|  |  | (*c*) If relevant, consider translating estimates of relative risk into absolute risk for a meaningful time period |

| Other analyses | 17 | Report other analyses done—eg analyses of subgroups and interactions, and sensitivity analyses  Subsequent analyses evaluated the risk of AMI stratified by AMI risk factors. Multiplicative interaction was tested to identify effect modification by stratifying factors.  Also, we assessed the dose-response relationship with risk of AMI through restricted cubic splines (smoothly joined piecewise cubic polynomial curves) providing greater flexibility to dose-response curves rather than assuming linearity. |
| --- | --- | --- |
| Discussion | | |
| Key results | 18 | Summarise key results with reference to study objectives  In this multinational case-control study nested in a new user NSAID cohort of more than 8 million persons, we assessed the association with acute myocardial infarction (AMI) for 28 individual NSAIDs with a total of 79,553 AMI cases. The study is unique in its kind. It capitalizes on the heterogeneity of prescribing patterns across countries, and the differences across databases, which allowed for analyses on more drugs than otherwise would be possible, while having common protocol, definitions, data transformation and analysis, which is a quantum leap better than meta-analyses of heterogeneous observational studies. |
| Limitations | 19 | Discuss limitations of the study, taking into account sources of potential bias or imprecision. Discuss both direction and magnitude of any potential bias  Since NSAID use was assessed through computerized prescriptions/dispensing, we could not capture over-the-counter NSAID use, this may lead to non-differential misclassification towards the null. Channeling of COX-2 inhibitors to high GI-risk patients in the initial marketing phase and the cardiovascular contra-indications after 2004 may have led to time-varying confounding by indication. First of all, we matched on calendar time both for the index date as well as cohort entry. Secondly past use of any NSAID was used as comparator, third we matched on database. In addition we adjusted for a large range of known risk factors for AMI. The matched and adjusted estimates were very similar, indicating that most of the potential confounding variables were time, sex and age-related and taken care of by the matching. Some residual confounding may remain due to inability to measure these confounders accurately (e.g. smoking), however because of the matching on database this is not likely to differ for cases and controls. |
| Interpretation | 20 | Give a cautious overall interpretation of results considering objectives, limitations, multiplicity of analyses, results from similar studies, and other relevant evidence  The risk of AMI risk varies with each individual agent as used in clinical practice in Europe for both traditional and COX-2 selective NSAIDs. The degree of COX-2 inhibition is not the unique feature determining the cardiovascular safety of NSAIDs as was initially advocated. The risk of AMI was increased for current use of several individual NSAIDs. The risk of AMI was highest for ketorolac, while the risk was increased by 30% for use of rofecoxib and diclofenac. Using higher doses showed higher risks of AMI. There appears to be an increased risk of AMI for several individual NSAIDs, rather than only a class effect for selective COX-2 inhibitors. This should prompt physicians to balance the cardiovascular risk of separate NSAIDs for each individual patient. Future studies should disentangle for each individual NSAID which risk factors are accurate predictors for not only cardiovascular diseases but also gastrointestinal diseases and create a decision model to balance the CV and gastrointestinal risk in order to prescribe the most ‘safe’ NSAID on an individual level. |
| Generalisability | 21 | Discuss the generalisability (external validity) of the study results  All six electronic health care databases contain a large number of patients and reflect the underlying general population. This study can be generalised to other Western European populations. |
| Other information | | |
| Funding | 22 | Give the source of funding and the role of the funders for the present study and, if applicable, for the original study on which the present article is based  The research leading to the results of this study has received funding from the European Community’s Seventh Framework Programme under grant agreement number 223495 - the SOS project. |

*Give information separately for cases and controls.

**Note:** An Explanation and Elaboration article discusses each checklist item and gives methodological background and published examples of transparent reporting. The STROBE checklist is best used in conjunction with this article (freely available on the Web sites of PLoS Medicine at http://www.plosmedicine.org/, Annals of Internal Medicine at http://www.annals.org/, and Epidemiology at http://www.epidem.com/). Information on the STROBE Initiative is available at http://www.strobe-statement.org.
