# Supplementary material for: Risk of acute myocardial infarction during use of individual NSAIDs: A nested case-control study from the SOS project
Source: PLoS One. 2018 Nov 1;13(11):e0204746. doi: 10.1371/journal.pone.0204746 (PMC6211656; doi:10.1371/journal.pone.0204746)
Supplement: S1 Table — (DOCX) [file pone.0204746.s002.docx]

**S1 Table: Characteristics of participating databases**

| **Database** | **GePaRD** | **IPCI** | **PHARMO** | **SISR** | **OSSIFF** | **THIN** |
| --- | --- | --- | --- | --- | --- | --- |
| **Country** | Germany | Netherlands | Netherlands | Italy | Italy | United Kingdom |
| **Type of Database** | Claims database | General practice database | Record linkage system | National Health Services registry (claims) | National Health Services registry (claims) | General practice database |
| **Study period** | 2005 - 2009 | 1999 – 2011 | 1999 – 2008 | 2000 – 2009 | 2002 – 2009 | 1999 – 2008 |
| **Coding system for diagnoses** | ICD-10-GM | ICPC and free text | ICD-9-CM | ICD-9-CM | ICD-9-CM | READ |
| **Outpatient hospital diagnoses** | Available | Available, as free text or codes | Available | Available | Available | Available |
| **Hospital discharge diagnoses** | Available | Available, as free text or codes | Available | Available | Available | Available |
| **Diagnostic procedures** | Available | Not available | Available | Available | Available | Available |
| **Laboratory tests** | Available ordering of the test | Available | Available, for a subset | Available | Available | Available |
| **Coding system for drugs** | ATC | ATC | ATC | ATC | ATC | BNF/ Multilex |
| **Date of prescription/dispensing** | Available | Available | Available | Available | Available | Available |
| **Dosing regimen** | Not available | Available | Available | Not available | Not available | Available |
| **Drug quantity** | Available | Available | Available | Available | Available | Available |

ICD-10-GM: International Classification of Diseases, 10^th^ Revision German Modified; ICD-9-CM: International Classification of Diseases, 9^th^ Revision Clinically Modified; ICPC: International Classification for Primary Care; ATC: Anatomical Therapeutic Chemical classification; BNF: British National Formulary
