# Supplementary material for: Risk of acute myocardial infarction during use of individual NSAIDs: A nested case-control study from the SOS project
Source: PLoS One. 2018 Nov 1;13(11):e0204746. doi: 10.1371/journal.pone.0204746 (PMC6211656; doi:10.1371/journal.pone.0204746)
Supplement: S2 Table — (DOCX) [file pone.0204746.s003.docx]

**S2 Table: NSAIDs included in the SOS project with annual prevalence of users per 100,000 person-years**

|  | **ATC** | **Substance** | **GePaRD** | **IPCI** | **PHARMO** | **SISR** | **OSSIFF** | **THIN** |
| --- | --- | --- | --- | --- | --- | --- | --- | --- |
| **M01AA** |  | **Butylpyrazolidines** |  |  |  |  |  |  |
|  | M01AA01 | Phenylbutazone | 39.9 | 2.8 | 3.0 |  |  | 0.04 |
|  | M01AA02 | Mofebutazone | 1.5 |  |  |  |  |  |
|  | M01AA03 | Oxyphenbutazone |  |  |  |  |  |  |
|  | M01AA05 | Clofezone |  |  |  |  |  |  |
|  | M01AA06 | Kebuzone |  |  |  |  |  |  |
| **M01AB** |  | **Acetic acid derivatives and related substances** | | |  |  |  |  |
|  | M01AB01 | Indometacin | 218.5 | 97.2 | 154.2 | 134.6 | 215.0 | 138.3 |
|  | M01AB02 | Sulindac |  | 9.1 | 23.4 | 1.0 | 1.3 | 3.5 |
|  | M01AB03 | Tolmetin |  | 0.2 | 1.3 |  |  |  |
|  | M01AB04 | Zomepirac |  |  |  |  |  |  |
|  | M01AB05 | Diclofenac | 8,092.4 | 5,482.9 | 4,373.6 | 1,666.9 | 2,342.2 | 3,379.8 |
|  | M01AB06 | Alclofenac |  |  |  |  |  |  |
|  | M01AB07 | Bumadizone |  |  |  |  |  |  |
|  | M01AB08 | Etodolac |  |  |  |  |  | 87.0 |
|  | M01AB09 | Lonazolac | 4.9 |  |  |  |  |  |
|  | M01AB10 | Fentiazac |  |  |  | 0.3 | 0.6 |  |
|  | M01AB11 | Acemetacin | 177.9 |  |  | 0.1 | 0.5 | 5.4 |
|  | M01AB12 | Difenpiramide |  |  |  |  |  |  |
|  | M01AB13 | Oxametacin |  |  |  |  |  |  |
|  | M01AB14 | Proglumetacin | 26.4 |  |  | 5.8 | 11.0 |  |
|  | M01AB15 | Ketorolac |  |  |  | 430.9 | 887.7 | 0.6 |
|  | M01AB16 | Aceclofenac | 77.7 | 17.5 | 43.1 | 411.9 | 411.9 | 12.2 |
|  | M01AB17 | Bufexamac |  |  |  |  |  |  |
|  | M01AB51 | Indometacin, combinations | |  |  |  | 8.9 |  |
|  | M01AB55 | Diclofenac, combinations | 194.5 | 1,085.1 | 1,094.7 | 72.3 | 126.9 | 359.6 |
| **M01AC** |  | **Oxicams** |  |  |  |  |  |  |
|  | M01AC01 | Piroxicam | 300.9 | 142.8 | 201.9 | 853.2 | 1535.2 | 78.0 |
|  | M01AC02 | Tenoxicam |  | 0.5 | 1.1 | 54.3 | 82.6 | 3.3 |
|  | M01AC04 | Droxicam |  |  |  |  |  |  |
|  | M01AC05 | Lornoxicam | 28.3 |  |  | 76.7 | 84.8 | 0.004 |
|  | M01AC06 | Meloxicam | 215.3 | 411.4 | 570.3 | 272.2 | 450.6 | 285.9 |
| **M01AE** |  | **Propionic acid derivatives** | |  |  |  |  |  |
|  | M01AE01 | Ibuprofen | 8,478.5 | 1,889.1 | 3,692.5 | 555.3 | 575.1 | 2,957.7 |
|  | M01AE02 | Naproxen | 192.4 | 1,649.9 | 2,384.1 | 184.8 | 298.5 | 692.3 |
|  | M01AE03 | Ketoprofen | 42.1 | 23.9 | 45.5 | 1,193.1 | 1,519.7 | 23.9 |
|  | M01AE04 | Fenoprofen |  |  |  |  |  | 0.9 |
|  | M01AE05 | Fenbufen |  |  |  |  |  | 4.4 |
|  | M01AE06 | Benoxaprofen |  |  |  |  |  |  |
|  | M01AE07 | Suprofen |  |  |  |  |  |  |
|  | M01AE08 | Pirprofen |  |  |  |  |  |  |
|  | M01AE09 | Flurbiprofen | 0.042 | 3.2 | 7.9 | 33.9 | 62.8 | 22.8 |
|  | M01AE10 | Indoprofen |  |  |  |  |  |  |
|  | M01AE11 | Tiaprofenic acid | 12.9 | 58.1 | 34.7 | 2.2 | 7.1 | 7.1 |
|  | M01AE12 | Oxaprozin | 0.7 |  |  | 62.7 | 91.5 |  |
|  | M01AE13 | Ibuproxam |  |  |  |  |  |  |
|  | M01AE14 | Dexibuprofen | 93.5 | 7.6 | 29.6 | 71.1 | 74.4 | 4.9 |
|  | M01AE15 | Flunoxaprofen |  |  |  |  |  |  |
|  | M01AE16 | Alminoprofen |  |  |  |  |  |  |
|  | M01AE17 | Dexketoprofen | 269.8 | 0.9 | 2.7 | 0.019 | 0.028 | 14.2 |
|  | M01AE18 | Naproxcinod |  |  |  |  |  |  |
|  | M01AE51 | Ibuprofen, combinations | |  |  |  | 0.009 | 10.2 |
|  | M01AE52 | Naproxen and esomeprazole | | 0.1 |  |  |  |  |
|  | M01AE53 | Ketoprofen, combinations | |  |  | 0.005 | 0.009 |  |
| **M01AG** |  | **Fenamates** |  |  |  |  |  |  |
|  | M01AG01 | Mefenamic acid | 0.003 |  |  | 1.2 | 1.0 | 397.0 |
|  | M01AG02 | Tolfenamic acid |  | 1.2 | 1.1 |  |  | 7.0 |
|  | M01AG03 | Flufenamic acid |  |  |  |  |  |  |
|  | M01AG04 | Meclofenamic acid |  |  |  | 0.005 |  |  |
| **M01AH** |  | **Coxibs** |  |  |  |  |  |  |
|  | M01AH01 | Celecoxib | 241.6 | 217.8 | 303.1 | 501.2 | 897.4 | 386.4 |
|  | M01AH02 | Rofecoxib | 0.3 | 186.0 | 576.5 | 311.3 | 738.3 | 301.1 |
|  | M01AH03 | Valdecoxib | 83.5 | 2.8 | 11.7 | 27.1 | 30.1 | 17.8 |
|  | M01AH04 | Parecoxib | 7.1 |  |  |  |  | 0.039 |
|  | M01AH05 | Etoricoxib | 412.9 | 406.1 | 284.5 | 382.6 | 434.8 | 155.6 |
|  | M01AH06 | Lumiracoxib | 14.6 |  |  |  |  | 3.2 |
| **M01AX** |  | **Other anti-inflammatory and antirheumatic agents, non-steroids** | | | | |  |  |
|  | M01AX01 | Nabumetone | 7.1 | 51.4 | 148.7 | 22.8 | 42.8 | 31.0 |
|  | M01AX02 | Niflumic acid |  |  |  | 0.01 | 5.4 |  |
|  | M01AX04 | Azapropazone |  | 10.5 | 15.8 |  |  | 7.6 |
|  | M01AX07 | Benzydamine |  |  |  |  |  |  |
|  | M01AX13 | Proquazone |  |  |  |  |  |  |
|  | M01AX17 | Nimesulide |  |  |  | 1,540.3 | 2,042.7 |  |
|  | M01AX18 | Feprazone |  |  |  |  |  |  |
|  | M01AX22 | Morniflumate |  |  |  | 0.1 | 2.5 |  |
|  | M01AX23 | Tenidap |  |  |  |  |  |  |
|  | M01AX68 | Feprazone, combinations | |  |  |  |  |  |
