# Supplementary material for: Risk of acute myocardial infarction during use of individual NSAIDs: A nested case-control study from the SOS project
Source: PLoS One. 2018 Nov 1;13(11):e0204746. doi: 10.1371/journal.pone.0204746 (PMC6211656; doi:10.1371/journal.pone.0204746)
Supplement: S3 Table — (DOCX) [file pone.0204746.s004.docx]

**S3 Table: List of diagnostic codes (plus related label) for acute myocardial infarction case identification in electronic medical records stratified by coding system**

| **ICD 9**  **(for PHARMO, OSSIFF, SISR)** | | | **ICD 10**  **(for GePaRD)** | | | **ICPC**  **(for IPCI)** | | | **READ**  **(for THIN)** | |
| --- | --- | --- | --- | --- | --- | --- | --- | --- | --- | --- |
| 410/ 410.9/ 410.90 | Acute myocardial infarction | | I21 | Acute myocardial infarction | | K75 | Acute myocardial infarction | | G30..00 | Acute myocardial infarction |
| 410.7 | Acute subendocardial myocardial infarction | | I21.4 | Acute subendocardial myocardial infarction | |  |  | | G307.00 | Acute subendocardial infarction |
| 410.07/ 410.71/ 410.72 | Acute myocardial infarction, subendocardial infarction | |  |  | |  |  | |  |  |
| 410/ 410.01/ 410.02 | Acute myocardial infarction of anterolateral wall | | I21.0 | Acute transmural myocardial infarction of anterior wall | |  |  | | G300.00 | Acute anterolateral infarction |
| 410.08 | Acute infarction of papillary muscle | |  |  | |  |  | | G30y100 | Acute papillary muscle infarction |
| 410.01 | Acute anteroseptal myocardial infarction | |  |  | |  |  | | G301100 | Acute anteroseptal infarction |
| 410.04 | Acute myocardial infarction of inferior wall | | I21.1 | Acute transmural myocardial infarction of inferior wall | |  |  | |  |  |
| 410.08 | Acute myocardial infarction of atrium | |  |  | |  |  | | G30y000 | Acute atrial infarction |
|  |  | |  |  | |  |  | | G30yz00 | Other acute myocardial infarction NOS |
|  |  | |  |  | |  |  | | G301000 | Acute anteroapical infarction |
| 410.08 | Acute myocardial infarction of septum | |  |  | |  |  | | G30y200 | Acute septal infarction |
| 410.03/ 410.3/ 410.31/ 410.32 | Acute myocardial infarction of inferoposterior wall | |  |  | |  |  | | G303.00 | Acute inferoposterior infarction |
| 410.02/ 410.2/ 410.21/ 410.22 | Acute myocardial infarction of inferolateral wall | |  |  | |  |  | | G302.00 | Acute inferolateral infarction |
| 410.01 | Acute myocardial infarction of anterior wall (disorder) | |  |  | |  |  | | G380.00 | Postoperative transmural myocardial infarction anterior wall |
| 410.01/ 410.1/ 410.11/ 410.12 | Acute myocardial infarction, of other anterior wall | |  |  | |  |  | |  |  |
| 410.04/ 410.4/ 410.41/ 410.42 | Acute myocardial infarction, of other inferior wall | |  |  | |  |  | | G308.00 | Inferior myocardial infarction NOS |
|  |  | |  |  | |  |  | |  |  |
|  |  | |  |  | |  |  | |  |  |
|  |  | |  |  | |  |  | |  |  |
| **ICD 9**  **(for PHARMO, OSSIFF, SISR)** | | | **ICD10**  **(for GePaRD)** | | | **ICPC**  **(for IPCI)** | | | **READ**  **(for THIN)** | |
|  | |  |  | |  |  | |  | G381.00 | Postoperative transmural myocardial infarction inferior wall |
| 410.05/ 410.5/ 410.51/ 410.52 | | Acute myocardial infarction, of other lateral wall |  | |  |  | |  | G305.00 | Lateral myocardial infarction NOS |
|  | |  |  | |  |  | |  |  |  |
| 410.08/ 410.8/ 410.81/ 410.82 | | Acute myocardial infarction, of other specified sites |  | |  |  | |  | G30z.00 | Acute Myocardial infarction NOS |
| 410.06/ 410.6/ 410.61/ 410.62 | | True posterior myocardial infarction |  | |  |  | |  | G306.00 | True posterior myocardial infarction |
|  | |  |  | |  |  | |  | G304.00 | Posterior myocardial infarction NOS |
| 410.91 | | Acute myocardial infarction, unspecified site, initial episode of care | I21.2/ I21.3 / I21.9 | | Acute transmural myocardial infarction of other sites/ Acute transmural myocardial infarction of unspecified site / Acute Myocardial infarction, unspecified |  | |  | Gyu34 | Acute transmural myocardial infarction of unspecified site |
| 410.92 | | Acute myocardial infarction, unspecified site, subsequent episode of care |  | |  |  | |  |  |  |
| 410 | | ST elevation (STEMI) and non-ST elevation (NSTEMI) myocardial infarction |  | |  |  | |  | G309.00 | Acute Q-wave infarct |
|  | |  |  | |  |  | |  | G307000 | Acute non-Q-wave infarction |
