# Supplementary material for: Risk of acute myocardial infarction during use of individual NSAIDs: A nested case-control study from the SOS project
Source: PLoS One. 2018 Nov 1;13(11):e0204746. doi: 10.1371/journal.pone.0204746 (PMC6211656; doi:10.1371/journal.pone.0204746)
Supplement: S4 Table — (DOCX) [file pone.0204746.s005.docx]

**S4 Table: Doses considered in the current study in the three databases that captured prescribed doses (THIN, IPCI, PHARMO).**

|  | **DDD value** | **PDD/DDD*** |  |  |  |
| --- | --- | --- | --- | --- | --- |
| **Current use of:** |  | **Cases** |  | **Controls** |  |
|  |  | **Median (Q1-Q3)** | **Mean (SD)** | **Median (Q1-Q3)** | **Mean (SD)** |
| Diclofenac | 100 mg | 1.5 (1.0-1.5) | 1.3 (0.4) | 1.5 (1.0-1.5) | 1.3 (0.5) |
| Fixed Combination of Diclofenac with misoprostol | 100 mg | 1.5 (1.0-1.5) | 1.3 (0.3) | 1.2 (1.0-1.5) | 1.2 (0.3) |
| Ibuprofen | 1200 mg | 1.0 (1.0-1.0) | 1.0 (0.4) | 1.0 (1.0-1.0) | 1.0 (0.3) |
| Naproxen | 500 mg | 2.0 (1.5-2.0) | 1.7 (0.5) | 2.0 (1.5-2.0) | 1.7 (0.6) |
| Meloxicam | 15 mg | 1.0 (0.5-1.0) | 0.8 (0.3) | 1.0 (0.5-1.0) | 0.8 (0.3) |
| Celecoxib | 200 mg | 1.0 (1.0-1.0) | 1.1 (0.5) | 1.0 (1.0-1.0) | 1.1 (0.4) |
| Rofecoxib | 25 mg | 1.0 (0.5-1.0) | 0.9 (0.3) | 1.0 (0.5-1.0) | 0.9 (0.3) |
| Etoricoxib | 60 mg | 1.5 (1.0-1.5) | 1.3 (0.5) | 1.5 (1.0-1.5) | 1.3 (0.5) |

PDD, prescribed daily dose; DDD, defined daily dose.

* PDD/DDD: the ratio of the prescribed daily dose with the defined daily dose as defined by the WHO.
