# Supplementary material for: Risk of acute myocardial infarction during use of individual NSAIDs: A nested case-control study from the SOS project
Source: PLoS One. 2018 Nov 1;13(11):e0204746. doi: 10.1371/journal.pone.0204746 (PMC6211656; doi:10.1371/journal.pone.0204746)
Supplement: S5 Table — (DOCX) [file pone.0204746.s006.docx]

**S5 Table:** **Association between current use of individual NSAIDs and the risk of AMI compared with past use of any NSAID in individual databases.**

|  | **Germany - GePaRD** | | | | **United Kingdom - THIN** | | | |
| --- | --- | --- | --- | --- | --- | --- | --- | --- |
|  | **Cases N = 9,930** | **Controls N = 957,016** | **ORmatched  (95% CI)** | **ORadj**  **(95% CI)** | **Cases N = 13,511** | **Controls N = 1,232,506** | **ORmatched  (95% CI)** | **ORadj**  **(95% CI)** |
| **Past Use of any NSAID** | 5,339 (53.8) | 542,672 (56.7) | 1 (ref) | 1 (ref) | 8377 (62.0) | 799,331 (64.9) | 1 (ref) | 1 (ref) |
| **Recent Use of any NSAID** | 3,138 (31.60) | 301,266 (31.48) | 1.02 (0.97-1.07) | 1.04 (0.98-1.09) | 3034 (22.46) | 62,979 (21.34) | **1.11 (1.06-1.17)** | **1.14 (1.10-1.19)** |
| **Current Use of:** |  |  |  |  |  |  |  |  |
| Aceclofenac | 6 (0.06) | 336 (0.04) | 1.64 (0.73-3.68) | 1.16 (0.46-2.94) | 4 (0.03) | 350 (0.03) |  |  |
| Acemetacin | 11 (0.11) | 1,028 (0.11) | 1.03 (0.57-1.87) | 0.95 (0.52-1.72) | 2 (0.01) | 127 (0.01) |  |  |
| Celecoxib | 13 (0.13) | 1,446 (0.15) | 0.86 (0.50-1.49) | 0.79 (0.46-1.37) | 183 (1.35) | 14,771 (1.20) | 1.10 (0.95-1.28) | 1.11 (0.95-1.28) |
| Dexibuprofen | 2 (0.02) | 279 (0.03) |  |  | 0 (0) | 46 (0.00) |  |  |
| Dexketoprofen | 8 (0.08) | 604 (0.06) | 1.32 (0.65-2.65) | 1.12 (0.54-2.33) | 1 (0.01) | 105 (0.01) |  |  |
| Diclofenac | 771 (7.76) | 64,062 (6.69) | **1.19 (1.10-1.29)** | **1.22 (1.13-1.33)** | 649 (4.80) | 54,019 (4.38) | **1.15 (1.06-1.25)** | **1.24 (1.14-1.34)** |
| Diclofenac, combinations | 19 (0.19) | 1,068 (0.11) | **1.63 (1.03-2.57)** | **1.68 (1.06-2.65)** | 147 (1.09) | 11,210 (0.91) | **1.19 (1.01-1.40)** | **1.22 (1.04-1.44)** |
| Etodolac |  |  |  |  | 37 (0.27) | 2,761 (0.22) | 1.22 (0.88-1.69) | 1.17 (0.85-1.63) |
| Etoricoxib | 47 (0.47) | 3,401 (0.36) | 1.31 (0.98-1.75) | 1.29 (0.96-1.72) | 53 (0.39) | 3,635 (0.29) | 1.31 (1.00-1.72) | 1.28 (0.97-1.68) |
| Flurbiprofen |  |  |  |  | 4 (0.03) | 260 (0.02) |  |  |
| Ibuprofen | 504 (5.08) | 35,593 (3.72) | **1.39 (1.26-1.53)** | **1.36 (1.23-1.50)** | 472 (3.49) | 40,291 (3.27) | 1.05 (0.96-1.16) | **1.15 (1.04-1.26)** |
| Indometacin | 24 (0.24) | 1,277 (0.13) | **1.79 (1.19-2.70)** | **1.71 (1.13-2.56)** | 47 (0.35) | 3,363 (0.27) | 1.31 (0.98-1.74) | **1.36 (1.02-1.82)** |
| Ketoprofen | 0 (0) | 238 (0.02) |  |  | 6 (0.04) | 701 (0.06) | 0.80 (0.36-1.78) | 0.94 (0.45-1.98) |
| Ketorolac |  |  |  |  | 0 (0) | 5 (0.00) |  |  |
| Lornoxicam | 1 (0.01) | 98 (0.01) |  |  |  |  |  |  |
| Mefenamic acid |  |  |  |  | 12 (0.09) | 910 (0.07) | 1.26 (0.71-2.23) | 1.18 (0.66-2.09) |
| Meloxicam | 19 (0.19) | 1,459 (0.15) | 1.24 (0.79-1.96) | 1.22 (0.78-1.93) | 128 (0.95) | 10,205 (0.83) | 1.12 (0.94-1.34) | 1.10 (0.92-1.31) |
| Nabumetone | 1 (0.01) | 66 (0.01) |  |  | 8 (0.06) | 839 (0.07) | 0.94 (0.48-1.83) | 0.94 (0.48-1.83) |
| Naproxen | 21 (0.21) | 1,009 (0.11) | **1.97 (1.28-3.05)** | **1.87 (1.20-2.89)** | 156 (1.15) | 13,389 (1.09) | 1.09 (0.93-1.28) | 1.12 (0.96-1.32) |
| Nimesulide |  |  |  |  |  |  |  |  |
| Oxaprozin |  |  |  |  |  |  |  |  |
| Piroxicam | 14 (0.14) | 1,639 (0.17) | 0.82 (0.48-1.38) | 0.84 (0.50-1.43) | 21 (0.16) | 1,654 (0.13) | 1.19 (0.77-1.82) | 1.16 (0.75-1.78) |
| Proglumetacin | 1 (0.01) | 137 (0.01) |  |  |  |  |  |  |
| Rofecoxib |  |  |  |  | 164 (1.21) | 11,225 (0.91) | **1.32 (1.12-1.54)** | **1.28 (1.09-1.5)** |
| Sulindac |  |  |  |  | 0 (0) | 94 (0.01) |  |  |
| Tenoxicam |  |  |  |  | 1 (0.01) | 110 (0.01) |  |  |
| Tiaprofenic acid | 0 (0) | 68 (0.01) |  |  | 3 (0.02) | 182 (0.01) |  |  |
| Valdecoxib | 0 (0) | 79 (0.01) |  |  | 6 (0.04) | 287 (0.02) | 1.87 (0.83-4.21) | 1.13 (0.39-3.29) |

ORmatched, matching criteria age, sex, indexdate (± 1 year), cohort entry (± 28 days) and database. ORadj, ORadjusted for backward selected confounders (for each database these may differ); 95% CI, 95 % Confidence Intervals.

Values in bold represent statistically significant estimates.

**S5 Table: Association between current use of individual NSAIDs and the risk of AMI compared with past use of any NSAID in individual databases (continued).**

|  | **The Netherlands - PHARMO** | | | | **The Netherlands - IPCI** | | | |
| --- | --- | --- | --- | --- | --- | --- | --- | --- |
|  | **Cases N = 9,974** | **Controls N = 896,907** | **ORmatched  (95% CI)** | **ORadj**  **(95% CI)** | **Cases N = 1,070** | **Controls N = 38,688** | **ORmatched  (95% CI)** | **ORadj**  **(95% CI)** |
| **Past Use of any NSAID** | 6,444 (64.6) | 618,802 (69.0) | 1 (ref) | 1 (ref) | 597 (55.8) | 19,025 (49.2) | 1 (ref) | 1 (ref) |
| **Recent Use of any NSAID** | 2316 (23.22) | 200,396 (22.34) | **1.09 (1.03-1.15)** | **1.11 (1.05-1.17)** | 341 (31.87) | 14,962 (38.67) | 1.01 (0.85-1.2) | 1.02 (0.85-1.22) |
| **Current Use of:** |  |  |  |  |  |  |  |  |
| Aceclofenac | 5 (0.05) | 272 (0.03) | 1.68 (0.69-4.06) | 1.52 (0.57-4.03) | 0 (0) | 1 (0.00) |  |  |
| Acemetacin |  |  |  |  |  |  |  |  |
| Celecoxib | 54 (0.54) | 2,553 (0.28) | **1.71 (1.30-2.25)** | **1.73 (1.32-2.27)** | 3 (0.28) | 94 (0.24) |  |  |
| Dexibuprofen | 4 (0.04) | 95 (0.01) |  |  | 0 (0) | 3 (0.01) |  |  |
| Dexketoprofen | 0 (0) | 14 (0.00) |  |  |  |  |  |  |
| Diclofenac | 420 (4.21) | 26,381 (2.94) | **1.41 (1.27-1.56)** | **1.44 (1.30-1.59)** | 58 (5.42) | 2,454 (6.34) | 1.07 (0.80-1.44) | 1.08 (0.79-1.47) |
| Diclofenac, combinations | 122 (1.22) | 7,748 (0.86) | **1.29 (1.08-1.55)** | **1.36 (1.13-1.63)** | 13 (1.21) | 554 (1.43) | 0.80 (0.45-1.43) | 0.75 (0.41-1.36) |
| Etodolac |  |  |  |  |  |  |  |  |
| Etoricoxib | 48 (0.48) | 2,413 (0.27) | **1.65 (1.24-2.20)** | **1.67 (1.25-2.23)** | 5 (0.47) | 250 (0.65) | 0.88 (0.36-2.18) | 0.94 (0.38-2.33) |
| Flurbiprofen | 2 (0.02) | 92 (0.01) |  |  | 0 (0) | 2 (0.01) |  |  |
| Ibuprofen | 207 (2.08) | 14,065 (1.57) | **1.33 (1.16-1.54)** | **1.43 (1.24-1.65)** | 18 (1.68) | 556 (1.44) | 1.28 (0.78-2.10) | 1.23 (0.74-2.06) |
| Indometacin | 23 (0.23) | 1,293 (0.14) | **1.60 (1.06-2.43)** | **1.61 (1.06-2.44)** | 1 (0.09) | 30 (0.08) |  |  |
| Ketoprofen | 1 (0.01) | 437 (0.05) |  |  | 1 (0.09) | 7 (0.02) |  |  |
| Ketorolac |  |  |  |  |  |  |  |  |
| Lornoxicam |  |  |  |  |  |  |  |  |
| Mefenamic acid |  |  |  |  |  |  |  |  |
| Meloxicam | 82 (0.82) | 5,396 (0.60) | **1.27 (1.02-1.58)** | 1.25 (1.00-1.55) | 8 (0.75) | 206 (0.53) | 1.33 (0.63-2.78) | 1.40 (0.65-3.02) |
| Nabumetone | 10 (0.10) | 903 (0.10) | 0.99 (0.53-1.86) | 1.03 (0.55-1.92) | 1 (0.09) | 13 (0.03) |  |  |
| Naproxen | 144 (1.44) | 9,838 (1.10) | **1.31 (1.10-1.55)** | **1.34 (1.13-1.59)** | 16 (1.50) | 463 (1.20) | 1.29 (0.76-2.20) | 1.27 (0.74-2.17) |
| Nimesulide |  |  |  |  |  |  |  |  |
| Oxaprozin |  |  |  |  |  |  |  |  |
| Piroxicam | 9 (0.09) | 1,199 (0.13) | 0.67 (0.35-1.30) | 0.65 (0.33-1.29) | 4 (0.37) | 25 (0.06) |  |  |
| Proglumetacin |  |  |  |  |  |  |  |  |
| Rofecoxib | 83 (0.83) | 4,974 (0.55) | **1.46 (1.17-1.83)** | **1.46 (1.17-1.83)** | 4 (0.37) | 43 (0.11) |  |  |
| Sulindac | 7 (0.07) | 300 (0.03) | 1.87 (0.88-3.96) | 1.41 (0.65-3.09) | 0 (0) | 6 (0.02) |  |  |
| Tenoxicam | 0 (0) | 11 (0.00) |  |  |  |  |  |  |
| Tiaprofenic acid | 1 (0.01) | 231 (0.03) |  |  | 2 (0.19) | 22 (0.06) |  |  |
| Valdecoxib | 0 (0) | 39 (0.00) |  |  |  |  |  |  |

ORmatched, matching criteria age, sex, indexdate (± 1 year), cohort entry (± 28 days) and database. ORadj, ORadjusted for backward selected confounders (for each database these may differ); 95% CI, 95 % Confidence Intervals.

**S5 Table: Association between current use of individual NSAIDs and the risk of AMI compared with past use of any NSAID in individual databases (continued).**

|  | **Italy - SISR** | | | | **Italy - OSSIFF** | | | |
| --- | --- | --- | --- | --- | --- | --- | --- | --- |
|  | **Cases N = 25,719** | **Controls N = 2,523,118** | **ORmatched  (95% CI)** | **ORadj**  **(95% CI)** | **Cases N = 19,349** | **Controls N = 1,840,368** | **ORmatched  (95% CI)** | **ORadj**  **(95% CI)** |
| **Past Use of any NSAID** | 12,265 (47.7) | 1,311,705 (52.0) | 1 (ref) | 1 (ref) | 10,732 (55.5) | 1,093,813 (59.4) | 1 (ref) | 1 (ref) |
| **Recent Use of any NSAID** | 9,128 (35.49) | 63,409 (34.22) | **1.08 (1.05-1.11)** | **1.05 (1.02-1.08)** | **5,939 (30.69)** | 38,476 (29.26) | **1.10 (1.06-1.14)** | **1.07 (1.03-1.10)** |
| **Current Use of:** |  |  |  |  |  |  |  |  |
| Aceclofenac | 141 (0.55) | 14,200 (0.56) | 0.97 (0.82-1.15) | 1.01 (0.85-1.19) | 58 (0.30) | 5,211 (0.28) | 1.05 (0.81-1.36) | 1.06 (0.81-1.37) |
| Acemetacin | 1 (0.00) | 4 (0.00) |  |  | 0 (0) | 19 (0.00) |  |  |
| Celecoxib | 366 (1.42) | 34,595 (1.37) | 1.05 (0.94-1.16) | 1.08 (0.97-1.20) | 267 (1.38) | 22,673 (1.23) | 1.13 (1.00-1.28) | 1.11 (0.98-1.26) |
| Dexibuprofen | 24 (0.09) | 1,539 (0.06) | **1.54 (1.03-2.30)** | 1.17 (0.75-1.84) | 11 (0.06) | 689 (0.04) | 1.52 (0.84-2.75) | 1.12 (0.57-2.22) |
| Dexketoprofen |  |  |  |  |  |  |  |  |
| Diclofenac | 720 (2.80) | 52,244 (2.07) | **1.38 (1.28-1.49)** | **1.39 (1.29-1.50)** | 446 (2.31) | 31,053 (1.69) | **1.39 (1.26-1.53)** | **1.36 (1.24-1.50)** |
| Diclofenac, combinations | 64 (0.25) | 4,398 (0.17) | **1.44 (1.12-1.84)** | **1.35 (1.05-1.74)** | 34 (0.18) | 2,945 (0.16) | 1.10 (0.79-1.55) | 1.09 (0.77-1.53) |
| Etodolac |  |  |  |  |  |  |  |  |
| Etoricoxib | 229 (0.89) | 19,631 (0.78) | **1.15 (1.01-1.31)** | **1.17 (1.02-1.33)** | 115 (0.59) | 8,148 (0.44) | **1.33 (1.10-1.60)** | **1.34 (1.11-1.61)** |
| Flurbiprofen | 15 (0.06) | 834 (0.03) | **1.78 (1.06-2.96)** | 1.14 (0.62-2.11) | 6 (0.03) | 784 (0.04) | 0.73 (0.33-1.63) | 0.93 (0.45-1.91) |
| Ibuprofen | 272 (1.06) | 20,588 (0.82) | **1.30 (1.15-1.47)** | **1.23 (1.09-1.39)** | 91 (0.47) | 8,126 (0.44) | 1.04 (0.85-1.29) | 1.02 (0.83-1.25) |
| Indometacin | 58 (0.23) | 3,722 (0.15) | **1.54 (1.18-1.99)** | **1.33 (1.02-1.75)** | 43 (0.22) | 2,104 (0.11) | **1.94 (1.43-2.63)** | **1.59 (1.16-2.20)** |
| Ketoprofen | 376 (1.46) | 30,916 (1.23) | **1.20 (1.09-1.34)** | **1.16 (1.05-1.29)** | 175 (0.90) | 15,670 (0.85) | 1.07 (0.92-1.24) | 1.04 (0.89-1.21) |
| Ketorolac | 148 (0.58) | 6,567 (0.26) | **2.24 (1.90-2.64)** | **2.00 (1.70-2.35)** | 124 (0.64) | 5,160 (0.28) | **2.31 (1.93-2.77)** | **2.14 (1.79-2.55)** |
| Lornoxicam | 22 (0.09) | 2,036 (0.08) | 1.05 (0.69-1.61) | 1.01 (0.66-1.55) | 17 (0.09) | 961 (0.05) | **1.67 (1.02-2.68)** | 1.21 (0.70-2.08) |
| Mefenamic acid | 0 (0) | 60 (0.00) |  |  | 0 (0) | 11 (0.00) |  |  |
| Meloxicam | 171 (0.66) | 13,501 (0.54) | **1.25 (1.07-1.45)** | **1.28 (1.10-1.49)** | 84 (0.43) | 8,039 (0.44) | 0.99 (0.80-1.23) | 1.01 (0.82-1.26) |
| Nabumetone | 14 (0.05) | 1,209 (0.05) | 1.15 (0.68-1.94) | 1.03 (0.60-1.79) | 12 (0.06) | 765 (0.04) | 1.52 (0.86-2.69) | 1.13 (0.59-2.16) |
| Naproxen | 87 (0.34) | 8,332 (0.33) | 1.03 (0.83-1.27) | 1.01 (0.81-1.24) | 62 (0.32) | 5,628 (0.31) | 1.06 (0.83-1.37) | 1.07 (0.83-1.38) |
| Nimesulide | 1,069 (4.16) | 85,349 (3.38) | **1.26 (1.18-1.34)** | **1.18 (1.11-1.26)** | 583 (3.01) | 48,113 (2.61) | **1.18 (1.08-1.28)** | **1.12 (1.04-1.23)** |
| Oxaprozin | 14 (0.05) | 1,740 (0.07) | 0.80 (0.47-1.35) | 0.93 (0.57-1.54) | 8 (0.04) | 969 (0.05) | 0.80 (0.40-1.60) | 0.92 (0.49-1.73) |
| Piroxicam | 324 (1.26) | 27,361 (1.08) | **1.17 (1.05-1.31)** | **1.17 (1.04-1.31)** | 264 (1.36) | 20,020 (1.09) | **1.27 (1.12-1.44)** | **1.28 (1.13-1.45)** |
| Proglumetacin | 6 (0.02) | 489 (0.02) | 1.19 (0.53-2.67) | 1.03 (0.42-2.49) | 4 (0.02) | 304 (0.02) |  |  |
| Rofecoxib | 180 (0.70) | 16,002 (0.63) | 1.13 (0.97-1.31) | 1.16 (1.00-1.35) | 259 (1.34) | 19,430 (1.06) | **1.31 (1.15-1.48)** | **1.27 (1.12-1.44)** |
| Sulindac | 3 (0.01) | 69 (0.00) |  |  | 1 (0.01) | 25 (0.00) |  |  |
| Tenoxicam | 21 (0.08) | 1,877 (0.07) | 1.10 (0.71-1.69) | 1.05 (0.67-1.63) | 10 (0.05) | 1,106 (0.06) | 0.86 (0.46-1.61) | 0.97 (0.53-1.78) |
| Tiaprofenic acid | 1 (0.00) | 124 (0.00) |  |  | 1 (0.01) | 83 (0.00) |  |  |
| Valdecoxib | 10 (0.04) | 1,299 (0.05) | 0.75 (0.40-1.41) | 0.91 (0.53-1.59) | 9 (0.05) | 455 (0.02) | 1.86 (0.96-3.61) | 1.14 (0.51-2.55) |

ORmatched, matching criteria age, sex, indexdate (± 1 year), cohort entry (± 28 days) and database. ORadj, ORadjusted for backward selected confounders (for each database these may differ); 95% CI, 95 % Confidence Intervals.

Values in bold represent statistically significant estimates.
