# Supplementary material for: Risk of acute myocardial infarction during use of individual NSAIDs: A nested case-control study from the SOS project
Source: PLoS One. 2018 Nov 1;13(11):e0204746. doi: 10.1371/journal.pone.0204746 (PMC6211656; doi:10.1371/journal.pone.0204746)
Supplement: S6 Table — (DOCX) [file pone.0204746.s007.docx]

**S6 Table: Characteristics of NSAID users by NSAID.**

|  | **naproxen** | | **celecoxib** | | **rofecoxib** | | **etoricoxib** | | **ibuprofen** | | **diclofenac** | | **indometacin** | | **ketorolac** | |
| --- | --- | --- | --- | --- | --- | --- | --- | --- | --- | --- | --- | --- | --- | --- | --- | --- |
|  | n | % | n | % | n | % | n | % | n | % | n | % | n | % | n | % |
| Diabetes Mellitus Type 2 | 2,836 | 7.2 | 6,827 | 8.9 | 4,363 | 8.3 | 3,356 | 8.8 | 10,511 | 8.7 | 19,817 | 8.5 | 1,007 | 8.4 | 1,309 | 10.9 |
| Heart Failure | 599 | 1.5 | 1,832 | 2.4 | 1,253 | 2.4 | 1,128 | 3.0 | 5,163 | 4.3 | 8,282 | 3.6 | 433 | 3.6 | 439 | 3.7 |
| Hyperlipidemia | 5,263 | 13.4 | 10,383 | 13.5 | 6,691 | 12.8 | 5,927 | 15.6 | 18,713 | 15.5 | 34,377 | 14.7 | 1,636 | 13.7 | 1,681 | 14.0 |
| Ischemic heart disease | 902 | 2.3 | 1,988 | 2.6 | 1,385 | 2.6 | 1,217 | 3.2 | 8,826 | 7.3 | 14,402 | 6.2 | 510 | 4.3 | 371 | 3.1 |
| Osteoarthritis | 2,260 | 5.8 | 5,431 | 7.1 | 3,757 | 7.2 | 2,504 | 6.6 | 15,572 | 12.9 | 24,457 | 10.5 | 803 | 6.7 | 301 | 2.5 |
| Smoking | 1,173 | 3.0 | 1,043 | 1.4 | 761 | 1.5 | 375 | 1.0 | 3,093 | 2.6 | 4,649 | 2.0 | 292 | 2.4 | 0 | 0.0 |
| Stroke | 209 | 0.5 | 538 | 0.7 | 316 | 0.6 | 296 | 0.8 | 2,484 | 2.1 | 3,704 | 1.6 | 138 | 1.2 | 90 | 0.7 |
| ACE-inhibitors and ATII antagonists | 6,653 | 17.0 | 18,371 | 23.9 | 11,725 | 22.4 | 8,896 | 23.4 | 24,773 | 20.5 | 46,652 | 20.0 | 2,692 | 22.5 | 3,133 | 26.1 |
| Low-dose aspirin | 7,320 | 18.7 | 15,134 | 19.6 | 10,541 | 20.1 | 6,840 | 18.0 | 19,889 | 16.5 | 34,560 | 14.8 | 2,203 | 18.4 | 2,329 | 19.4 |
| Beta-blockers | 5,218 | 13.3 | 9074 | 11.8 | 6394 | 12.2 | 5,206 | 13.7 | 21,632 | 17.9 | 37,033 | 15.9 | 1,991 | 16.6 | 1,397 | 11.6 |
| Calcium channel blockers | 5,423 | 13.9 | 16,592 | 21.5 | 11,407 | 21.8 | 7,651 | 20.1 | 18,782 | 15.6 | 35,826 | 15.4 | 2,198 | 18.3 | 2,741 | 22.8 |
| Diuretics | 6,324 | 16.2 | 16,661 | 21.6 | 11,337 | 21.7 | 6,642 | 17.5 | 22,913 | 19.0 | 36,035 | 15.4 | 2,330 | 19.4 | 2,439 | 20.3 |
| Glucocorticoids | 2,363 | 6.0 | 5,544 | 7.2 | 3,175 | 6.1 | 3,047 | 8.0 | 6,759 | 5.6 | 14,494 | 6.2 | 1,035 | 8.6 | 1,480 | 12.3 |
| Lipid modifying agents | 7,233 | 18.5 | 11,947 | 15.5 | 7,670 | 14.6 | 6,705 | 17.7 | 21,773 | 18.0 | 40,031 | 17.2 | 1,988 | 16.6 | 1,670 | 13.9 |
| Nitrates | 2,444 | 6.2 | 7,288 | 9.5 | 5,535 | 10.6 | 2,540 | 6.7 | 7,669 | 6.3 | 13,435 | 5.8 | 896 | 7.5 | 1,446 | 12.0 |
| Oral contraceptives | 318 | 0.8 | 97 | 0.1 | 110 | 0.2 | 70 | 0.2 | 510 | 0.4 | 724 | 0.3 | 24 | 0.2 | 26 | 0.2 |
| Hypertensive Drugs | 3,400 | 8.7 | 13,335 | 17.3 | 8,038 | 15.4 | 7,162 | 18.9 | 13,947 | 11.5 | 31,338 | 13.4 | 1,690 | 14.1 | 2,255 | 18.8 |
| Antiplatelets | 992 | 2.5 | 3,264 | 4.2 | 1,955 | 3.7 | 1,676 | 4.4 | 2,953 | 2.4 | 6,110 | 2.6 | 389 | 3.2 | 732 | 6.1 |
| Hormone Therapy | 853 | 2.2 | 1,533 | 2.0 | 1,211 | 2.3 | 753 | 2.0 | 3,478 | 2.9 | 5,387 | 2.3 | 163 | 1.4 | 150 | 1.2 |
