# Supplementary material for: Risk of acute myocardial infarction during use of individual NSAIDs: A nested case-control study from the SOS project
Source: PLoS One. 2018 Nov 1;13(11):e0204746. doi: 10.1371/journal.pone.0204746 (PMC6211656; doi:10.1371/journal.pone.0204746)
Supplement: S7 Table — (DOCX) [file pone.0204746.s008.docx]

**S7 Table: Effect modification by important (proxy) risk factors for AMI on the matched pooled dataset.**

|  | **Sex** | | | | | **Age** | | | | |
| --- | --- | --- | --- | --- | --- | --- | --- | --- | --- | --- |
| **NSAID** | **Females** | | **Males** | |  | **<= 60 Years** | | **> 60 Years** | |  |
| **Current use of:** | **Cases**  **N** | **ORadj**  **(95% CI)** | **Cases**  **N** | **ORadj**  **(95% CI)** | **P-value interaction** | **Cases**  **N** | **ORadj**  **(95% CI)** | **Cases**  **N** | **ORadj**  **(95% CI)** | **P-value interaction** |
| Aceclofenac | 123 | 1.01 (0.73-1.41) | 91 | 1.22 (0.87-1.69) | 0.447 | **38** | **2.26 (1.41-3.60)** | **176** | **0.99 (0.76-1.29)** | **0.016** |
| Acemetacin | 3 | 0.92 (0.35-2.46) | 11 | 1.08 (0.57-2.04) | 0.238 | 5 | 1.24 (0.48-3.21) | 9 | 0.96 (0.50-1.82) | 0.306 |
| Celecoxib | 529 | 1.13 (1.00-1.27) | 357 | 1.17 (1.03-1.34) | 0.833 | 112 | 1.36 (1.08-1.71) | 774 | 1.12 (1.02-1.23) | 0.124 |
| Dexibuprofen | 20 | 0.95 (0.43-2.12) | 21 | 1.28 (0.63-2.62) | 0.083 | 4 | 0.93 (0.25-3.41) | 37 | 1.13 (0.63-2.03) | 0.375 |
| Dexketoprofen | 0 | NA | 9 | 1.14 (0.53-2.47) | - | 3 | 0.99 (0.32-3.14) | 6 | 1.03 (0.42-2.50) | 0.844 |
| Diclofenac | **1,201** | **1.38 (1.28-1.49)** | **1,863** | **1.25 (1.18-1.32)** | **0.008** | 879 | 1.39 (1.28-1.50) | 2,185 | 1.25 (1.19-1.32) | 0.069 |
| Diclofenac combinations | 186 | 1.27 (1.08-1.50) | 213 | 1.28 (1.11-1.48) | 0.745 | 79 | 1.34 (1.06-1.69) | 320 | 1.22 (1.08-1.38) | 0.549 |
| Etodolac | 17 | 1.10 (0.66-1.83) | 20 | 1.09 (0.69-1.72) | 0.823 | 9 | 1.24 (0.63-2.44) | 28 | 1.07 (0.73-1.58) | 0.644 |
| Etoricoxib | 261 | 1.24 (1.03-1.48) | 236 | 1.46 (1.23-1.72) | 0.133 | 74 | 1.41 (1.09-1.84) | 423 | 1.31 (1.14-1.50) | 0.524 |
| Flurbiprofen | 14 | 1.05 (0.49-2.27) | 13 | 0.94 (0.39-2.22) | 0.362 | 3 | 1.06 (0.24-4.75) | 24 | 1.00 (0.53-1.85) | 0.825 |
| Ibuprofen | 660 | 1.30 (1.19-1.43) | 904 | 1.24 (1.15-1.34) | 0.369 | 403 | 1.31 (1.17-1.46) | 1,161 | 1.22 (1.14-1.31) | 0.277 |
| Indometacin | 66 | 1.48 (1.06-2.08) | 130 | 1.56 (1.28-1.90) | 0.471 | 36 | 1.19 (0.82-1.72) | 160 | 1.74 (1.44-2.10) | 0.076 |
| Ketoprofen | 291 | 1.01 (0.82-1.24) | 268 | 1.00 (0.81-1.23) | 0.800 | 78 | 0.72 (0.46-1.12) | 481 | 1.04 (0.89-1.22) | 0.143 |
| Ketorolac | 138 | 1.94 (1.49-2.52) | 134 | 2.16 (1.69-2.77) | 0.924 | 33 | 1.55 (0.93-2.58) | 239 | 2.19 (1.81-2.66) | 0.153 |
| Lornoxicam | 24 | 1.07 (0.51-2.20) | 16 | 1.19 (0.53-2.68) | 0.488 | 4 | 0.93 (0.17-5.16) | 36 | 1.17 (0.67-2.07) | 0.388 |
| Mefenamic acid | 8 | 1.05 (0.49-2.25) | 4 | 1.01 (0.36-2.83) | 0.815 | 6 | 1.12 (0.48-2.58) | 6 | 1.02 (0.43-2.44) | 0.996 |
| Meloxicam | 267 | 1.08 (0.92-1.26) | 225 | 1.17 (1.00-1.36) | 0.498 | 69 | 1.09 (0.83-1.42) | 423 | 1.11 (0.98-1.25) | 0.930 |
| Nabumetone | 29 | 1.10 (0.68-1.79) | 17 | 0.96 (0.56-1.65) | 0.419 | 3 | 0.57 (0.18-1.83) | 43 | 1.15 (0.79-1.67) | 0.063 |
| Naproxen | 190 | 1.19 (1.01-1.40) | 296 | 1.24 (1.09-1.40) | 0.689 | **165** | **1.44 (1.22-1.70)** | **321** | **1.09 (0.96-1.24)** | **0.013** |
| Nimesulide | 837 | 1.10 (0.98-1.24) | 815 | 1.17 (1.04-1.32) | 0.772 | 226 | 1.32 (1.05-1.65) | 1,426 | 1.11 (1.01-1.21) | 0.273 |
| Oxaprozin | 12 | 0.96 (0.40-2.30) | 10 | 0.95 (0.39-2.30) | 0.980 | 2 | 0.95 (0.16-5.52) | 20 | 0.95 (0.48-1.86) | 0.951 |
| Piroxicam | 342 | 1.31 (1.12-1.54) | 294 | 1.18 (1.00-1.39) | 0.396 | 86 | 1.19 (0.90-1.57) | 550 | 1.22 (1.08-1.39) | 0.730 |
| Proglumetacin | 7 | 0.99 (0.27-3.63) | 4 | 1.03 (0.29-3.65) | 0.699 | 1 | 1.03 (0.12-8.81) | 10 | 1.00 (0.37-2.71) | 0.849 |
| Rofecoxib | 378 | 1.24 (1.09-1.40) | 312 | 1.40 (1.23-1.60) | 0.256 | 80 | 1.34 (1.04-1.72) | 610 | 1.30 (1.18-1.43) | 0.790 |
| Sulindac | 6 | 1.04 (0.38-2.78) | 5 | 0.99 (0.32-3.07) | 0.589 | 2 | 1.02 (0.13-8.15) | 9 | 1.03 (0.46-2.29) | 0.991 |
| Tenoxicam | 17 | 0.95 (0.43-2.08) | 15 | 1.03 (0.44-2.37) | 0.494 | 3 | 0.91 (0.17-4.76) | 29 | 0.99 (0.53-1.82) | 0.651 |
| Tiaprofenic acid | 5 | 1.06 (0.36-3.15) | 3 | 0.95 (0.35-2.57) | 0.260 | 1 | 0.88 (0.18-4.18) | 7 | 1.04 (0.45-2.38) | 0.418 |
| Valdecoxib | 15 | 1.13 (0.50-2.51) | 10 | 1.08 (0.43-2.76) | 0.728 | 3 | 1.12 (0.22-5.70) | 22 | 1.13 (0.59-2.16) | 0.846 |
| Recent use of any NSAID | 10,378 | 1.07 (1.03-1.11) | 13,518 | 1.09 (1.06-1.12) | 0.609 | **5,663** | **1.17 (1.12-1.21)** | **18,233** | **1.05 (1.03-1.08)** | **<0.001** |
| Current use of any NSAID* |  | 1.25 (1.2-1.31) |  | 1.27 (1.22-1.31) | 0.894 |  | **1.35 (1.28-1.43)** |  | **1.23 (1.19-1.26)** | **0.001** |

ORadj, ORadjusted for backward selected confounders. Bold numbers indicate significant interaction-terms.

* Current use of any NSAID is a combination category of all individual NSAIDs that were classified as current use.

|  | **Co morbid ischemic heart disease** | | | | | **Current use of low-dose aspirin** | | | | |
| --- | --- | --- | --- | --- | --- | --- | --- | --- | --- | --- |
| **NSAID** | **Yes** | | **No** | |  | **Yes** | | **No** | |  |
| **Current use of:** | **Cases**  **N** | **ORadj**  **(95% CI)** | **Cases**  **N** | **ORadj**  **(95% CI)** | **P-value interaction** | **Cases**  **N** | **ORadj**  **(95% CI)** | **Cases**  **N** | **ORadj**  **(95% CI)** | **P-value interaction** |
| Aceclofenac | 9 | 1.39 (0.34-5.79) | 205 | 1.12 (0.88-1.43) | 0.727 | 65 | 1.14 (0.72-1.80) | 149 | 1.08 (0.81-1.42) | 0.591 |
| Acemetacin | 3 | 0.79 (0.27-2.30) | 11 | 1.04 (0.53-2.02) | 0.405 | 4 | NA | 10 | 0.97 (0.53-1.78) | - |
| Celecoxib | 57 | 0.89 (0.57-1.38) | 829 | 1.16 (1.06-1.28) | 0.305 | 227 | 1.00 (0.84-1.19) | 659 | 1.20 (1.08-1.33) | 0.070 |
| Dexibuprofen | 1 | NA | 40 | 1.15 (0.65-2.00) | - | 13 | 1.13 (0.38-3.40) | 28 | 1.07 (0.57-2.01) | 0.821 |
| Dexketoprofen | 4 | NA | 5 | 1.01 (0.40-2.57) | - | 3 | 1.13 (0.19-6.65) | 6 | 1.00 (0.45-2.22) | 0.368 |
| Diclofenac | 360 | 1.13 (0.98-1.30) | 2,704 | 1.32 (1.26-1.38) | 0.952 | 759 | 1.19 (1.08-1.31) | 2,305 | 1.32 (1.25-1.39) | 0.708 |
| Diclofenac combinations | 24 | 1.11 (0.62-2.02) | 375 | 1.26 (1.13-1.41) | 0.560 | 125 | 1.19 (0.97-1.46) | 274 | 1.27 (1.12-1.45) | 0.918 |
| Etodolac | 0 | NA | 37 | 1.16 (0.82-1.63) | - | 14 | 1.19 (0.67-2.12) | 23 | 1.08 (0.70-1.66) | 0.847 |
| Etoricoxib | **24** | **0.57 (0.31-1.05)** | **473** | **1.39 (1.23-1.58)** | **0.013** | 133 | 1.35 (1.05-1.74) | 364 | 1.34 (1.17-1.55) | 0.792 |
| Flurbiprofen | 0 | NA | 27 | 1.02 (0.57-1.82) | - | 10 | 1.02 (0.36-2.94) | 17 | 1.00 (0.50-2.02) | 0.813 |
| Ibuprofen | **256** | **1.32 (1.13-1.55)** | **1,308** | **1.23 (1.16-1.31)** | **0.001** | 414 | 1.18 (1.04-1.33) | 1150 | 1.27 (1.19-1.36) | 0.602 |
| Indometacin | 25 | 1.97 (1.16-3.34) | 171 | 1.53 (1.28-1.83) | 0.217 | 53 | 1.64 (1.17-2.32) | 143 | 1.59 (1.31-1.94) | 0.976 |
| Ketoprofen | 42 | 1.35 (0.62-2.96) | 517 | 0.99 (0.85-1.16) | 0.504 | 175 | 1.01 (0.76-1.35) | 384 | 0.98 (0.82-1.16) | 0.523 |
| Ketorolac | 29 | 1.42 (0.52-3.91) | 243 | 2.03 (1.68-2.45) | 0.670 | 87 | 2.80 (1.97-3.98) | 185 | 1.75 (1.40-2.19) | 0.074 |
| Lornoxicam | 4 | NA | 36 | 1.17 (0.67-2.03) | - | 14 | 0.98 (0.31-3.06) | 26 | 1.17 (0.62-2.21) | 0.261 |
| Mefenamic acid | 0 | NA | 12 | 1.07 (0.56-2.01) | - | 3 | NA | 9 | 1.05 (0.50-2.23) | - |
| Meloxicam | 36 | 0.87 (0.49-1.56) | 456 | 1.11 (0.99-1.25) | 0.839 | 141 | 0.94 (0.76-1.16) | 351 | 1.20 (1.05-1.37) | 0.051 |
| Nabumetone | 2 | NA | 44 | 1.07 (0.74-1.54) | - | 16 | 1.25 (0.69-2.26) | 30 | 0.96 (0.61-1.52) | 0.119 |
| Naproxen | 30 | 1.95 (1.17-3.23) | 456 | 1.18 (1.06-1.31) | 0.222 | 146 | 1.21 (1.00-1.47) | 340 | 1.18 (1.05-1.34) | 0.614 |
| Nimesulide | 108 | 1.03 (0.58-1.82) | 1,544 | 1.15 (1.06-1.26) | 0.336 | 523 | 0.99 (0.82-1.18) | 1,129 | 1.19 (1.08-1.31) | 0.085 |
| Oxaprozin | 2 | NA | 20 | 0.96 (0.50-1.84) | - | 6 | 0.95 (0.27-3.35) | 16 | 0.96 (0.46-2.00) | 0.770 |
| Piroxicam | 41 | 0.86 (0.49-1.51) | 595 | 1.22 (1.08-1.37) | 0.616 | 163 | 0.92 (0.71-1.20) | 473 | 1.31 (1.15-1.48) | 0.078 |
| Proglumetacin | 1 | NA | 10 | 1.00 (0.38-2.63) | - | 1 | 1.02 (0.09-11.22) | 10 | 1.00 (0.37-2.75) | 0.746 |
| Rofecoxib | 53 | 1.20 (0.73-1.96) | 637 | 1.32 (1.21-1.45) | 0.408 | 198 | 1.20 (1.01-1.42) | 492 | 1.37 (1.23-1.53) | 0.291 |
| Sulindac | 1 | NA | 10 | 1.02 (0.49-2.16) | - | 4 | NA | 7 | 1.01 (0.36-2.81) | - |
| Tenoxicam | 4 | NA | 28 | 0.92 (0.50-1.69) | - | 4 | NA | 28 | 1.02 (0.53-1.95) | - |
| Tiaprofenic acid | 1 | NA | 7 | 1.01 (0.46-2.21) | - | 2 | NA | 6 | 0.98 (0.43-2.25) | - |
| Valdecoxib | 0 | NA | 25 | 1.15 (0.62-2.13) | - | 2 | NA | 23 | 1.20 (0.61-2.36) | - |
| Recent use of any NSAID | 2,119 | 1.05 (0.96-1.14) | 21,177 | 1.09 (1.06-1.11) | 0.257 | 6,444 | 1.07 (1.02-1.12) | 17,452 | 1.09 (1.06-1.11) | 0.132 |
| Current use of any NSAID* |  | 1.14 (1.02-1.27) |  | 1.27 (1.24-1.31) | 0.337 |  | **1.16 (1.10-1.23)** |  | **1.28 (1.24-1.33)** | **0.041** |

ORadj, ORadjusted for backward selected confounders. Bold numbers indicate significant interaction-terms.

* Current use of any NSAID is a combination category of all individual NSAIDs that were classified as current use.

|  | **Use of lipid lowering drugs** | | | | | **Calendar Year** | | | | |
| --- | --- | --- | --- | --- | --- | --- | --- | --- | --- | --- |
| **NSAID** | **Yes** | | **No** | |  | **≤2004** | | **>2004** | |  |
| **Current Use of:** | **Cases N** | **ORadj (95% CI)** | **Cases N** | **ORadj (95% CI)** | **P-value interaction** | **Cases N** | **ORadj (95% CI)** | **Cases N** | **ORadj (95% CI)** | **P-value interaction** |
| Aceclofenac | 38 | 1.31 (0.78-2.20) | 176 | 1.07 (0.82-1.41) | 0.395 |  | 1.20 (0.78-1.84) |  | 0.99 (0.74-1.33) | 0.1387 |
| Acemetacin | 4 | NA | 10 | 0.99 (0.52-1.89) | - |  | 1.03 (0.12-8.75) |  | 0.94 (0.53-1.67) | 0.1685 |
| Celecoxib | 187 | 1.08 (0.89-1.31) | 699 | 1.15 (1.04-1.28) | 0.879 |  | 1.09 (0.98-1.22) |  | 1.24 (1.06-1.45) | 0.1268 |
| Dexibuprofen | 12 | 1.14 (0.39-3.35) | 29 | 1.06 (0.56-1.99) | 0.721 |  | 1.06 (0.15-7.42) |  | 1.11 (0.64-1.93) | 0.1354 |
| Dexketoprofen | 3 | NA | 6 | 1.01 (0.43-2.39) | - |  | 1.00 (0.15-6.57) |  | 1.06 (0.50-2.23) | 0.8469 |
| Diclofenac | **680** | **1.10 (1.00-1.21)** | **2,384** | **1.35 (1.29-1.42)** | **0.002** |  | 1.27 (1.18-1.37) |  | 1.31 (1.24-1.38) | 0.5647 |
| Diclofenac, combinations | 99 | 1.19 (0.95-1.49) | 300 | 1.30 (1.15-1.48) | 0.301 |  | 1.27 (1.09-1.48) |  | 1.24 (1.07-1.45) | 0.9872 |
| Etodolac | 16 | 1.28 (0.75-2.17) | 21 | 1.03 (0.66-1.60) | 0.654 |  | 0.94 (0.51-1.72) |  | 1.33 (0.91-1.97) | 0.1188 |
| Etoricoxib | 113 | 1.44 (1.13-1.84) | 384 | 1.35 (1.17-1.55) | 0.886 |  | 1.27 (0.98-1.65) |  | 1.35 (1.17-1.55) | 0.9032 |
| Flurbiprofen | 6 | 0.88 (0.23-3.36) | 21 | 1.02 (0.53-1.96) | 0.641 |  | 1.06 (0.51-2.19) |  | 0.91 (0.36-2.29) | 0.2622 |
| Ibuprofen | 376 | 1.16 (1.02-1.31) | 1,188 | 1.30 (1.22-1.39) | 0.115 |  | 1.23 (1.12-1.36) |  | 1.27 (1.18-1.37) | 0.5502 |
| Indometacin | 40 | 1.49 (1.04-2.15) | 156 | 1.64 (1.36-1.99) | 0.412 |  | 1.46 (1.13-1.87) |  | 1.65 (1.32-2.08) | 0.7266 |
| Ketoprofen | 108 | 0.65 (0.44-0.97) | 451 | 1.06 (0.90-1.24) | 0.287 |  | 1.07 (0.88-1.30) |  | 0.91 (0.73-1.14) | 0.3850 |
| Ketorolac | 61 | 2.02 (1.28-3.20) | 211 | 1.91 (1.56-2.35) | 0.117 |  | 1.62 (1.27-2.08) |  | 2.76 (2.10-3.63) | 0.0113 |
| Lornoxicam | 9 | 1.33 (0.40-4.49) | 31 | 1.07 (0.56-2.01) | 0.369 |  | 1.02 (0.01-84.87) |  | 1.25 (0.74-2.10) | 0.2239 |
| Mefenamic acid | 1 | NA | 11 | 1.08 (0.53-2.18) | - |  | 1.01 (0.47-2.18) |  | 1.07 (0.39-2.94) | 0.6187 |
| Meloxicam | 113 | 1.00 (0.79-1.26) | 379 | 1.19 (1.05-1.36) | 0.101 |  | 1.17 (1.00-1.37) |  | 1.07 (0.91-1.25) | 0.5839 |
| Nabumetone | 7 | 0.79 (0.36-1.75) | 39 | 1.10 (0.74-1.65) | 0.271 |  | 1.08 (0.69-1.70) |  | 0.96 (0.53-1.72) | 0.5087 |
| Naproxen | 134 | 1.17 (0.96-1.43) | 352 | 1.20 (1.07-1.35) | 0.846 |  | 1.14 (0.98-1.32) |  | 1.27 (1.11-1.46) | 0.2329 |
| Nimesulide | 361 | 1.13 (0.92-1.39) | 1,291 | 1.14 (1.03-1.25) | 0.314 |  | 1.09 (0.98-1.22) |  | 1.20 (1.05-1.38) | 0.2445 |
| Oxaprozin | 6 | 0.92 (0.20-4.23) | 16 | 0.97 (0.48-1.95) | 0.563 |  | 0.95 (0.41-2.19) |  | 0.96 (0.38-2.43) | 0.7890 |
| Piroxicam | 119 | 1.22 (0.92-1.62) | 517 | 1.26 (1.11-1.43) | 0.933 |  | 1.24 (1.07-1.43) |  | 1.21 (1.01-1.46) | 0.9356 |
| Proglumetacin | 3 | NA | 8 | 0.98 (0.36-2.68) | - |  | 0.98 (0.23-4.12) |  | 1.06 (0.34-3.35) | 0.3752 |
| Rofecoxib | 146 | 1.21 (0.98-1.49) | 544 | 1.36 (1.22-1.50) | 0.632 |  | 1.31 (1.20-1.43) |  | NA | - |
| Sulindac | 4 | NA | 7 | 1.02 (0.39-2.66) | - |  | 1.00 (0.41-2.47) |  | 1.04 (0.28-3.92) | 0.7048 |
| Tenoxicam | 3 | NA | 29 | 0.99 (0.53-1.83) | - |  | 1.02 (0.53-1.94) |  | 0.91 (0.27-3.06) | 0.2879 |
| Tiaprofenic acid | 1 | NA | 7 | 1.03 (0.45-2.34) | - |  | 1.07 (0.43-2.64) |  | 0.92 (0.26-3.23) | 0.1785 |
| Valdecoxib | 5 | 1.05 (0.28-3.94) | 20 | 1.12 (0.55-2.26) | 0.645 |  | 1.11 (0.49-2.51) |  | 1.09 (0.43-2.74) | 0.6754 |
| Recent use of any NSAID | 5,380 | 1.04 (1.00-1.09) | 18,516 | 1.09 (1.06-1.12) | 0.471 |  | 1.07 (1.03-1.11) |  | 1.09 (1.06-1.12) | 0.5595 |
| Current use of any NSAID* |  | **1.13 (1.07-1.20)** |  | **1.30 (1.26-1.34)** | **0.002** |  | 1.23 (1.18-1.28) |  | 1.29 (1.24-1.33) | 0.1289 |

ORa, ORadjusted for backward selected confounders. Bold numbers indicate significant interaction-terms.

* Current use of any NSAID is a combination category of all individual NSAIDs that were classified as current use.
