# Supplementary material for: Risk of acute myocardial infarction during use of individual NSAIDs: A nested case-control study from the SOS project
Source: PLoS One. 2018 Nov 1;13(11):e0204746. doi: 10.1371/journal.pone.0204746 (PMC6211656; doi:10.1371/journal.pone.0204746)
Supplement: S8 Table — (DOCX) [file pone.0204746.s009.docx]

**S8 Table: NSAIDs included in the SOS project with mean duration of use in days per person**

|  | **ATC** | **Substance** | **GePaRD** | **IPCI** | **PHARMO** | **SISR** | **OSSIFF** | **THIN** |
| --- | --- | --- | --- | --- | --- | --- | --- | --- |
| **Mean follow-up time in the NSAID cohort** | | | **923** | **723** | **1,376** | **1,284** | **1,493** | **1,331** |
| **M01AA** |  | **Butylpyrazolidines** |  |  |  |  |  |  |
|  | M01AA01 | Phenylbutazone | 12 | 313.8 | 483.7 |  |  | 123.4 |
|  | M01AA02 | Mofebutazone | 1.7 |  |  |  |  |  |
| **M01AB** |  | **Acetic acid derivatives and related substances** | | |  |  |  |  |
|  | M01AB01 | Indometacin | 67.9 | 83.1 | 110.1 | 26.4 | 23.3 | 164.6 |
|  | M01AB02 | Sulindac |  | 63.3 | 76 | 89.6 | 64.2 | 259.8 |
|  | M01AB03 | Tolmetin |  | 394.8 | 132.9 |  |  |  |
|  | M01AB05 | Diclofenac | 54.2 | 41.6 | 62.3 | 31.8 | 35.3 | 123.7 |
|  | M01AB08 | Etodolac |  |  |  |  |  | 276.4 |
|  | M01AB09 | Lonazolac | 68 |  |  |  |  |  |
|  | M01AB10 | Fentiazac |  |  |  | 134.6 | 136.3 |  |
|  | M01AB11 | Acemetacin | 74.5 |  |  | 48.4 | 86.9 | 362.9 |
|  | M01AB14 | Proglumetacin | 65.6 |  |  | 100.8 | 91 |  |
|  | M01AB15 | Ketorolac |  |  |  | 9.6 | 10.2 | 46.7 |
|  | M01AB16 | Aceclofenac | 56.7 | 64.2 | 62.8 | 38.7 | 37.9 | 253.8 |
|  | M01AB55 | Diclofenac combinations | 65.5 | 58.8 | 70.1 | 70 | 69.9 | 203.8 |
| **M01AC** |  | **Oxicams** |  |  |  |  |  |  |
|  | M01AC01 | Piroxicam | 53.3 | 80.5 | 83.6 | 33.3 | 37.1 | 238.3 |
|  | M01AC02 | Tenoxicam |  | 157.8 | 382.5 | 34 | 36 | 453.7 |
|  | M01AC05 | Lornoxicam | 68.6 |  |  | 34.1 | 35.7 | 10 |
|  | M01AC06 | Meloxicam | 68.7 | 71.9 | 90.5 | 60.5 | 57.8 | 188.6 |
| **M01AE** |  | **Propionic acid derivatives** | |  |  |  |  |  |
|  | M01AE01 | Ibuprofen | 29.8 | 37.9 | 43.9 | 35.9 | 37.2 | 71.2 |
|  | M01AE02 | Naproxen | 58.6 | 49.4 | 69.7 | 75.3 | 83.7 | 148.8 |
|  | M01AE03 | Ketoprofen | 71.1 | 213.8 | 210.3 | 27.4 | 28.4 | 385.4 |
|  | M01AE04 | Fenoprofen |  |  |  |  |  | 252.1 |
|  | M01AE05 | Fenbufen |  |  |  |  |  | 405.4 |
|  | M01AE09 | Flurbiprofen | 4.5 | 167.7 | 199.3 | 28.4 | 36.5 | 136.7 |
|  | M01AE11 | Tiaprofenic acid | 69.9 | 53.7 | 95.6 | 61.2 | 39.8 | 356 |
|  | M01AE12 | Oxaprozin | 49.7 |  |  | 33.4 | 33.1 |  |
|  | M01AE14 | Dexibuprofen | 43.4 | 75.1 | 49.4 | 21.1 | 22.6 | 73.8 |
|  | M01AE17 | Dexketoprofen | 10.6 | 10.6 | 25.8 | 10.5 | 32.2 | 44.9 |
|  | M01AE51 | Ibuprofen combinations |  |  |  |  | 8 | 111 |
|  | M01AE52 | Naproxen and esomeprazole |  | 12.7 |  |  |  |  |
|  | M01AE53 | Ketoprofen combinations |  |  |  | 50 | 30 |  |
| **M01AG** |  | **Fenamates** |  |  |  |  |  |  |
|  | M01AG01 | Mefenamic acid | 25 |  |  | 34.3 | 27.6 | 59.2 |
|  | M01AG02 | Tolfenamic acid |  | 109.5 | 331.1 |  |  | 34.1 |
|  | M01AG04 | Meclofenamic acid |  |  |  | 332.3 |  |  |
| **M01AH** |  | **Coxibs** |  |  |  |  |  |  |
|  | M01AH01 | Celecoxib | 84.9 | 95 | 95.2 | 76.9 | 76.9 | 183 |
|  | M01AH02 | Rofecoxib | 31.6 | 64.4 | 77.6 | 55.8 | 91.7 | 124.8 |
|  | M01AH03 | Valdecoxib | 78.5 | 107.3 | 76.8 | 58.6 | 55.7 | 116.4 |
|  | M01AH04 | Parecoxib | 4.8 |  |  |  |  | 14.8 |
|  | M01AH05 | Etoricoxib | 66.3 | 94.3 | 100.9 | 68.7 | 65.3 | 166.2 |
|  | M01AH06 | Lumiracoxib | 39 |  |  |  |  | 92.5 |
| **M01AX** |  | **Other anti-inflammatory and antirheumatic agents, non-steroids** | | | | |  |  |
|  | M01AX01 | Nabumetone | 35.3 | 125.1 | 95.2 | 61.5 | 61.1 | 330.8 |
|  | M01AX02 | Niflumic acid |  |  |  | 40 | 12.7 |  |
|  | M01AX04 | Azapropazone |  | 59.1 | 105.3 |  |  | 148.2 |
|  | M01AX07 | Benzydamine |  |  |  |  |  |  |
|  | M01AX17 | Nimesulide |  |  |  | 58.4 | 69.6 |  |
|  | M01AX22 | Mornifluminate |  |  |  | 20.8 | 13.6 |  |
