# Supplementary material for: Risk of acute myocardial infarction during use of individual NSAIDs: A nested case-control study from the SOS project
Source: PLoS One. 2018 Nov 1;13(11):e0204746. doi: 10.1371/journal.pone.0204746 (PMC6211656; doi:10.1371/journal.pone.0204746)
Supplement: S9 Table — (DOCX) [file pone.0204746.s010.docx]

**S9 Table:** Association between current use of individual NSAIDs and the risk of AMI compared with past use of any NSAID before index date in individual databases in patients with a low cardiovascular risk profile.

|  | IPCI | PHARMO | GEPARD | OSSIFF | THIN | UNIMIB |
| --- | --- | --- | --- | --- | --- | --- |
|  | ORadj (95%CI) | ORadj (95%CI) | ORadj (95%CI) | ORadj (95%CI) | ORadj (95%CI) | ORadj (95%CI) |
| **Past use of any NSAID** | 1 (ref) | 1 (ref) | 1 (ref) | 1 (ref) | 1 (ref) | 1 (ref) |
| **Current use of:** |  |  |  |  |  |  |
| Aceclofenac |  | 0.66 (0.09-4.72) |  | 1.02 (0.68-1.53) |  | 1.38 (1.07-1.82) |
| Acemetacin |  |  |  |  |  |  |
| Celecoxib |  | 2.17 (1.47-3.22) | 1.36 (0.18-10.05) | 1.20 (0.98-1.47) | 1.20 (0.92-1.56) | 1.31 (1.06-1.61) |
| Dexibuprofen |  | 4.70 (1.45-15.27) |  | 0.58 (0.14-2.33) |  | 0.93 (0.34-2.51) |
| Dexketoprofen |  |  |  |  |  |  |
| Diclofenac | 1.18 (0.84-1.67) | 1.37 (1.19-1.58) | 1.70 (1.27-2.28) | 1.41 (1.22-1.63) | 1.30 (1.15-1.47) | 1.26 (1.09-1.45) |
| Diclofenac combinations | 0.37 (0.13-1.02) | 1.23 (0.94-1.63) |  | 0.83 (0.46-1.51) | 1.18 (0.89-1.58) | 0.84 (0.45-1.58) |
| Etodolac |  |  |  |  | 1.60 (0.97-2.64) |  |
| Etoricoxib | 1.35 (0.53-3.42) | 2.07 (1.41-3.04) | 1.10 (0.27-4.49) | 1.18 (0.86-1.62) | 1.40 (0.89-2.18) | 1.33 (1.04-1.70) |
| Flurbiprofen |  |  |  | 0.28 (0.04-1.99) |  | 1.30 (0.41-4.10) |
| Ibuprofen | 0.98 (0.50-1.93) | 1.33 (1.11-1.60) | 1.70 (1.18-2.45) | 1.24 (0.92-1.67) | 1.20 (1.03-1.40) | 1.29 (1.00-1.66) |
| Indometacin |  | 2.18 (1.32-3.60) | 1.36 (0.18-10.11) | 1.69 (1.08-2.66) | 1.31 (0.81-2.10) | 1.89 (1.23-2.91) |
| Ketoprofen |  |  |  | 0.99 (0.78-1.24) | 0.41 (0.06-2.95) | 1.24 (1.03-1.50) |
| Ketorolac |  |  |  | 1.66 (1.25-2.22) |  | 1.63 (1.17-2.27) |
| Lornoxicam |  |  |  | 1.28 (0.53-3.13) |  | 0.96 (0.43-2.18) |
| Mefenamic acid |  |  |  |  | 1.44 (0.67-3.12) |  |
| Meloxicam | 1.52 (0.60-3.89) | 1.27 (0.89-1.81) |  | 0.76 (0.51-1.12) | 1.47 (1.11-1.95) | 1.15 (0.83-1.61) |
| Nabumetone |  | 0.93 (0.34-2.51) |  | 0.81 (0.20-3.29) | 0.31 (0.04-2.21) | 1.19 (0.38-3.78) |
| Naproxen | 0.91 (0.44-1.88) | 1.10 (0.86-1.41) | 3.75 (0.50-28.09) | 1.47 (1.05-2.06) | 1.14 (0.89-1.45) | 1.09 (0.73-1.64) |
| Nimesulide |  |  |  | 1.18 (1.03-1.35) |  | 1.07 (0.93-1.23) |
| Oxaprozin |  |  |  | 1.17 (0.48-2.85) |  | 0.66 (0.21-2.08) |
| Piroxicam |  | 0.97 (0.43-2.18) | 3.03 (0.91-10.07) | 1.25 (1.04-1.51) | 1.10 (0.52-2.34) | 1.14 (0.92-1.41) |
| Proglumetacin |  |  |  |  |  |  |
| Rofecoxib |  | 1.75 (1.25-2.45) |  | 1.58 (1.29-1.95) | 1.47 (1.11-1.96) | 1.00 (0.70-1.44) |
| Sulindac |  | 1.94 (0.26-14.51) |  |  |  |  |
| Tenoxicam |  |  |  | 0.89 (0.33-2.39) |  | 1.20 (0.53-2.71) |
| Tiaprofenic acid |  |  |  |  |  |  |
| Valdecoxib |  |  |  | 0.73 (0.10-5.28) | 1.84 (0.44-7.65) | 0.69 (0.17-2.81) |

* a low cardiovascular risk profile was defined as: patients free of ischemic heart disease, stroke, diabetes and without use of aspirin, lipid modifying agents, nitrates, beta blockers, diuretics and calcium channel blockers
