# Supplementary material for: Risk of acute myocardial infarction during use of individual NSAIDs: A nested case-control study from the SOS project
Source: PLoS One. 2018 Nov 1;13(11):e0204746. doi: 10.1371/journal.pone.0204746 (PMC6211656; doi:10.1371/journal.pone.0204746)
Supplement: S1 Fig — (DOCX) [file pone.0204746.s011.docx]

**S1 Figure: Flowchart of source population and study population per database**

* In IPCI cancer subjects have been excluded from the incident NSAID users cohort in order to do case validation of outcomes. therefore numbers in the flowchart at the level of ‘Incident NSAID users’ and ‘Cancer free at NSAID cohort entry’ for IPCI are similar.
