# Supplementary material for: Risk of acute myocardial infarction during use of individual NSAIDs: A nested case-control study from the SOS project
Source: PLoS One. 2018 Nov 1;13(11):e0204746. doi: 10.1371/journal.pone.0204746 (PMC6211656; doi:10.1371/journal.pone.0204746)
Supplement: S2 Fig — (DOCX) [file pone.0204746.s012.docx]

**S2 Figure: Relation between degree of inhibition of whole blood COX-2**[**^1^**](#_ENREF_1) **and risk of AMI for individual NSAIDs in the three databases that included doses (THIN. IPCI. PHARMO).**

R2=0.4491

* the OR for dose 0.8-1.2 pdd/ddd was chosen for plotting in this figure.

1. Garcia Rodriguez LA. Tacconelli S. Patrignani P. Role of dose potency in the prediction of risk of myocardial infarction associated with nonsteroidal anti-inflammatory drugs in the general population. *J Am Coll Cardiol* 2008;52(20):1628-36.
