# Supplementary material for: Risk of acute myocardial infarction during use of individual NSAIDs: A nested case-control study from the SOS project
Source: PLoS One. 2018 Nov 1;13(11):e0204746. doi: 10.1371/journal.pone.0204746 (PMC6211656; doi:10.1371/journal.pone.0204746)
Supplement: S3 Fig — (DOCX) [file pone.0204746.s013.docx]

**S3 Figure: Dose response estimated by fractional polynomial regression for diclofenac**

AppendixFigure 3A shows the estimated dose-response curves for current use of diclofenac.

The dose-response relationship is modeled through restricted cubic splines. implemented in a conditional logistic regression model. A cubic spline is a smoothly joined piecewise cubic polynomial curve. In particular. in cubic spline models the observed range of exposure is divided into different categories. and within each category a third-order polynomials is fitted. Cubic spline models provide great flexibility for fitting dose-response curves to data. To obtain a regular pattern even in the most extreme ranges of the dose. restricted cubic splines were considered. Restricted cubic splines have a linear trend outside the most extreme knots. In the present analysis three knots were considered corresponding to 0 DDD. 0.8 DDDs and 1.2 DDDs. This choice of knots allows to represent a dose-response relationship potentially non-linear in the dose range from 0 DDD to 1.2 DDD. and assumed to be linear for doses over 1.2 DDDs

The bold blue line represents the polynomial curve with 1^st^ degree term with corresponding 95% confidence intervals.

Dose on the x-asis represents the dose as was estimated from the prescribing regimen and strength. The dose is calculated by dividing the prescribed dose by the recommended daily dose (DDD).

1. Diclofenac


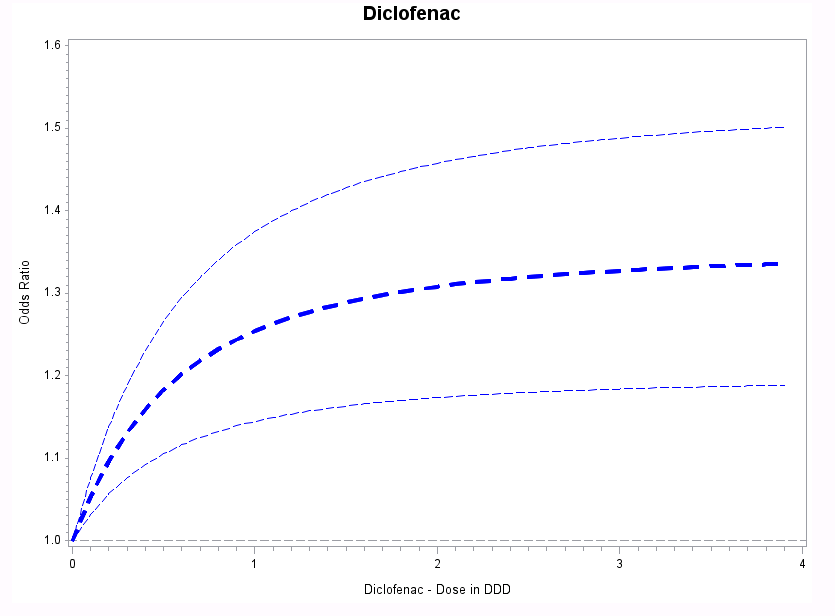


Diclofenac – Dose in PDD/DDD
