# Supplementary material for: Risk of acute myocardial infarction during use of individual NSAIDs: A nested case-control study from the SOS project
Source: PLoS One. 2018 Nov 1;13(11):e0204746. doi: 10.1371/journal.pone.0204746 (PMC6211656; doi:10.1371/journal.pone.0204746)
Supplement: S4 Fig — (DOCX) [file pone.0204746.s014.docx]

**S4 Figure: Adjusted risk estimates of AMI in current users of individual NSAIDs for duration of use in three databases pooled (THIN. IPCI. PHARMO) using short duration (7-29 days) as reference group.**
